# Protocol for a randomized controlled trial examining multilevel prediction of response to behavioral activation and exposure-based therapy for generalized anxiety disorder

**DOI:** 10.1186/s13063-019-3802-9

**Published:** 2020-01-06

**Authors:** J. Santiago, E. Akeman, N. Kirlic, A. N. Clausen, K. T. Cosgrove, T. J. McDermott, B. Mathis, M. Paulus, M. G. Craske, J. Abelson, C. Martell, K. Wolitzky-Taylor, J. Bodurka, W. K. Thompson, Robin L. Aupperle

**Affiliations:** 1 Laureate Institute for Brain Research, 6655 South Yale Avenue, Tulsa, OK 74136 USA; 2 School of Community Medicine, University of Tulsa, Tulsa, OK USA; 3 Department of Psychology, University of Tulsa, Tulsa, OK USA; 4 VA Mid-Atlantic Mental Illness Research, Education and Clinical Center, Durham, NC USA; 5 Duke University Brain Imaging and Analysis Center, Durham, NC USA; 6 Psychology, Psychiatry and Biobehavioral Sciences, University of California, Los Angeles, Los Angeles, CA USA; 7 Department of Psychiatry, University of Michigan, Ann Arbor, MI USA; 8 Department of Psychological and Brain Sciences, University of Massachusetts–Amherst, Amherst, MA USA; 9 Stephenson School of Biomedical Engineering, The University of Oklahoma, Norman, OK USA; 10 Family Medicine and Public Health, University of California, San Diego, San Diego, CA USA

**Keywords:** Generalized anxiety disorder, Depression, Behavioral activation, Exposure therapy, Cognitive behavioral therapy, Functional magnetic resonance imaging

## Abstract

**Background:**

Only 40–60% of patients with generalized anxiety disorder experience long-lasting improvement with gold standard psychosocial interventions. Identifying neurobehavioral factors that predict treatment success might provide specific targets for more individualized interventions, fostering more optimal outcomes and bringing us closer to the goal of “personalized medicine.” Research suggests that reward and threat processing (approach/avoidance behavior) and cognitive control may be important for understanding anxiety and comorbid depressive disorders and may have relevance to treatment outcomes. This study was designed to determine whether approach-avoidance behaviors and associated neural responses moderate treatment response to exposure-based versus behavioral activation therapy for generalized anxiety disorder.

**Methods/design:**

We are conducting a randomized controlled trial involving two 10-week group-based interventions: exposure-based therapy or behavioral activation therapy. These interventions focus on specific and unique aspects of threat and reward processing, respectively. Prior to and after treatment, participants are interviewed and undergo behavioral, biomarker, and neuroimaging assessments, with a focus on approach and avoidance processing and decision-making. Primary analyses will use mixed models to examine whether hypothesized approach, avoidance, and conflict arbitration behaviors and associated neural responses at baseline moderate symptom change with treatment, as assessed using the Generalized Anxiety Disorder–7 item scale. Exploratory analyses will examine additional potential treatment moderators and use data reduction and machine learning methods.

**Discussion:**

This protocol provides a framework for how studies may be designed to move the field toward neuroscience-informed and personalized psychosocial treatments. The results of this trial will have implications for approach-avoidance processing in generalized anxiety disorder, relationships between levels of analysis (i.e., behavioral, neural), and predictors of behavioral therapy outcome.

**Trial registration:**

The study was retrospectively registered within 21 days of first participant enrollment in accordance with FDAAA 801 with ClinicalTrials.gov, NCT02807480. Registered on June 21, 2016, before results.

## Background

Anxiety disorders are the most common mental health problem in the United States [1], and generalized anxiety disorder (GAD) is the most common anxiety disorder in primary care, with a lifetime prevalence rate of 6% [2]. It is a debilitating disorder leading to significant individual and socioeconomic burden with estimated annual costs of over $1500 per patient [2, 3]. Its prognosis is poor, with only 58% of cases experiencing remission within 2 years [4]. GAD is accompanied by major depressive disorder (MDD) in approximately 72% of cases, while MDD is accompanied by GAD in 48% of cases [5]. GAD in those with depression predicts poorer clinical outcomes and increased suicidal ideation compared with those with depression alone [6].

Psychotropic medications (e.g., selective serotonin reuptake inhibitors [SSRIs]) and psychotherapeutic interventions (e.g., cognitive behavioral therapy [CBT]) are both effective evidence-based treatments for GAD [7, 8]. However, only 40–60% of patients experience improvement with these treatments [9, 10], and 15–25% of those who improve relapse within 1 year [9]. This creates both clinical and socioeconomic challenges because these treatments are costly and time-consuming [11]. By identifying cognitive, behavioral, or neural factors that predict outcomes and can perhaps be targeted in an individualized fashion, we can move toward personalized approaches that assign each patient to the optimal treatment for them.

The National Institute of Mental Health (NIMH) Research Domain Criteria (RDoC) initiative seeks to improve mental health assessment and treatment by enhancing understanding of basic psychological domains across multiple levels of analysis (e.g., neural systems, physiology, behavior) [12]. Ideally, enhanced understanding of these domains will inform personalized treatment approaches. For example, if individual profiles of functioning across positive valence (e.g., approaching reward), negative valence (e.g., avoiding threat), or cognitive function (e.g., cognitive control) domains are identifiable, they could predict likelihood of success for various treatment approaches [13]. To achieve this eventual goal of “personalized medicine” [14], it is necessary to conduct clinical trials assessing these multilevel domains of function, randomize participants to comparator treatments, and examine common and unique predictors of treatment outcome.

Symptom severity, chronicity of symptoms, and comorbidity have been identified as potential predictors of GAD treatment response [15], but these findings do not provide specific targets for improving treatment effectiveness. The neurocognitive investigation of GAD has focused on enhanced negative affect and threat detection, as well as contradictory theories of either inadequate top-down prefrontal cortex (PFC) regulation (e.g., of amygdala) or PFC overactivation supporting maladaptive cognitive strategies (e.g., worry) [16]. There have been few neuroimaging studies examining predictors of psychosocial treatment response with GAD [17]. One study indicated that greater frontal, temporal, and insular activation during emotion reappraisal may predict better CBT response [18], but no GAD study to date has examined the use of neuroimaging to uniquely predict outcomes of two effective but divergent interventions.

Anxiety disorders have been hypothesized to arise from conflicting motivations to approach or avoid anxiogenic situations that also contain potential gains, leading to chronic distress, uncertainty, and use of maladaptive coping mechanisms (i.e., avoidance, worry) [19, 20]. Animal paradigms thought to be relevant for GAD rely heavily on approach-avoidance conflict (AAC) (such as Vogel or Geller-Seifter conflict test, in which a behavior is associated with both rewards, e.g., food pellet, and punishment, e.g., shock) [21, 22]. We developed a human AAC task [23, 24] for use in functional magnetic resonance imaging (fMRI) work, and we have shown that approach behavior was linked to caudate and anterior cingulate cortex activation, whereas difficulties arbitrating conflict were linked to self-reported anxiety and dorsolateral prefrontal cortex (dlPFC) activation. Given that GAD treatments typically focus on decreasing cognitive and behavioral avoidance [25], the ability to successfully arbitrate conflict (make decisions to approach or avoid) could theoretically contribute to propensity for treatment response. MDD has been associated with dysfunction in both reward and threat processing [26], and MDD treatments often focus on increasing meaningful engagement in rewarding or pleasurable activities [27]. Thus, approach-avoidance processing is likely important in understanding treatment for the clinical presentation of GAD with or without comorbid depression.

**Fig. 1:**
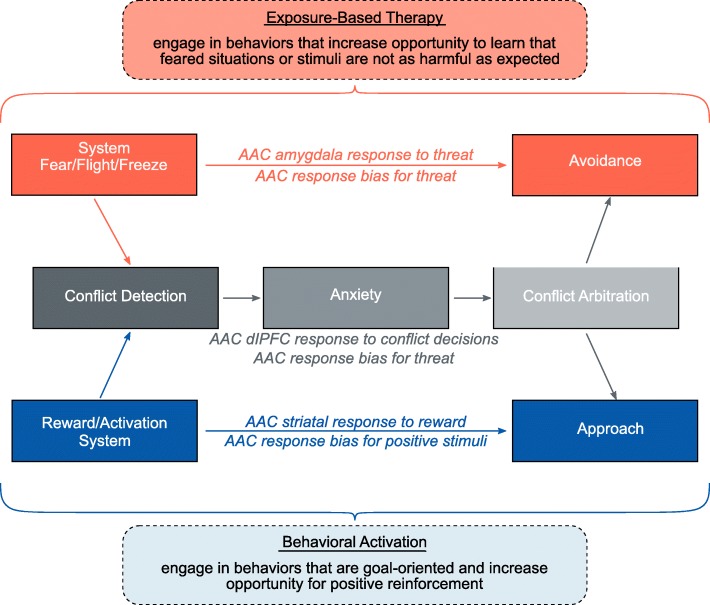
Approach-avoidance conflict model that provided the bases for the current study protocol, aims, and hypotheses. As illustrated, both the fear or avoidance system and the reward/approach system are considered important in eliciting conflict and anxiety. Conflict arbitration requires appropriate balancing of both approach and avoidance drives. In the current protocol, approach and avoidance behaviors are defined by approach-avoidance test (AAT) bias scores; conflict arbitration is defined by reaction time during the approach-avoidance conflict (AAC) trials. For brain responses, we focus on the AAC task and extract percentage signal change (PSC) from *a priori* regions of interest: (1) approach: left caudate (reward versus no-reward outcome), (2) avoidance: right amygdala (negative versus positive affective outcome), and (3) conflict: right dorsolateral prefrontal cortex (dlPFC; conflict versus nonconflict decisions). Exposure-based therapy was included as a treatment that primarily targets avoidance or threat processes, whereas behavioral activation was included as a treatment that primarily targets approach or reward systems

Herein we present the protocol of an ongoing study designed to address the need for identifying moderators of GAD treatment response. This protocol explores multilevel moderators (self-report, behavioral, and neuroimaging) of response to exposure therapy (EXP) [25, 28] versus behavioral activation (BA) [29]. These interventions were chosen due to their (1) likelihood of being effective for GAD and (2) specific and unique functional targets relating to RDoC domains (i.e., avoidance/threat targeted by EXP versus approach/reinforcement processing targeted by BA; see Fig. 1). We focused on the following aims:

1. Examine relationships among multilevel approach-avoidance behavior and neural responses and baseline GAD symptom severity
2. Examine how multilevel approach-avoidance behavior and neural responses moderate individualized response to exposure-based therapy versus BA for GAD
3. Identify the changes in approach-avoidance processes that relate to EXP- versus BA-elicited symptom improvement

## Methods/design

This protocol was written using the Standard Protocol Items: Recommendations for Interventional Trials (SPIRIT) guidelines, and the SPIRIT checklist is provided in Additional file 2. The protocol is part of an ongoing, randomized (two-condition), single-center (Laureate Institute for Brain Research [LIBR], Tulsa, OK, USA), controlled trial examining multilevel predictors of response to EXP versus BA for GAD. The study is currently recruiting and is registered with ClinicalTrials.gov (identifier NCT02807480; registration date June 21, 2016). No amendments have been made to the protocol since original submission to ClinicalTrials.gov. The study is funded by the National Institute of Mental Health (grant K23MH108707; Robin L. Aupperle [RLA], principal investigator [PI]) and the William K. Warren Foundation. Interventions include 10 weeks of manualized, group-based BA or EXP therapy. Groups of 8-10 participants are randomized altogether to a therapy group (randomization conducted in blocks of 4; sequence generated by RLA). Participants are kept blind to their intervention condition until completion of all baseline assessments; outcome assessors are partially blinded (see further description in Additional file 1). Primary predictor variables of interest are assessed using the approach-avoidance task (AAT) and the AAC task, whereas the primary outcome measure is the GAD-7. Secondary outcome measures include the Sheehan Disability Scale [30], NIH Patient-Reported Outcomes Measurement Information System anxiety and depression scales [31], Beck Depression Inventory-II (BDI-II) [32], and Penn State Worry Questionnaire [33].

**Fig. 2:**
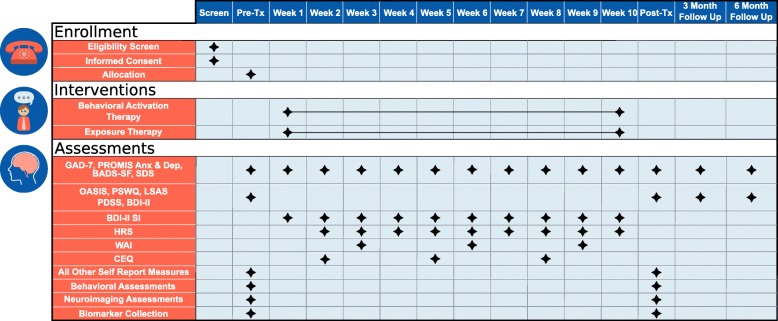
Schedule of enrollment, interventions, and assessments. This figure displays the assessments or interventions completed for screening, pretreatment, weekly during completion of therapy, posttreatment, and 3- and 6-month follow-up. Participants are randomized in groups of 8–10 to complete either behavioral activation or exposure-based therapy and are blinded to which intervention they will receive until after completion of all pretreatment assessments. *Tx* treatment, *BDI-II SI* Beck Depression Inventory suicidal ideation item, *GAD-7* Generalized Anxiety Disorder 7-item scale, *PROMIS Anx & Dep* Patient-Reported Outcomes Measurement Information System anxiety and depression scales, *BADS-SF* Behavioral Activation for Depression Scale–Short Form, *SDS* Sheehan Disability Scale, *HRS* Homework Rating Scale, *OASIS* Overall Anxiety Severity and Impairment Scale, *PSWQ* Penn State Worry Questionnaire, *LSAS* Liebowitz Social Anxiety Scale, *PDSS* Panic Disorder Severity Scale, *WAI* Working Alliance Inventory, *CEQ* Credibility/Expectancy Questionnaire

The overall study protocol is presented in Fig. 2. Screening assessments confirm exclusion and inclusion criteria for the study; baseline assessment includes self-report, behavioral, biological, and neuroimaging assessments. After baseline assessment, individuals are randomized to EXP or BA treatment, during which weekly self-report symptom measures are obtained. After treatment, participants repeat baseline assessments. Self-report symptom measures are repeated at 3 and 6 months following treatment. Research is conducted ethically in accordance with the World Medical Association Declaration of Helsinki. Research personnel trained in human subject research obtain written informed consent from each participant prior to completing any research procedures. The consent form for the study is included in Additional file 5.

### Participants

Projected enrollment is 100 treatment-seeking individuals meeting *Diagnostic and Statistical Manual of Mental Disorders, Fifth Edition* (DSM-5), GAD criteria [34] over a 5-year period (April 2016–April 2021), recruited from community mental health clinics and the general community through electronic and print advertisements. Participants must be 18–55 years old, have sufficient English proficiency to understand study procedures, and meet DSM-5 criteria per the Mini International Neuropsychiatric Interview (MINI 7.0) for GAD and score > 7 on the Overall Anxiety Severity and Impairment Scale [35]. Participants are excluded for the following reasons: (1) severe depressive symptoms (Patient Health Questionnaire-9 score > 17) and/or suicidal ideation with intent or plan, to decrease safety concerns and help ensure that GAD was the primary cause of impairment; (2) history of substance use disorder in the past 6 months; (3) meeting diagnostic criteria for psychotic, bipolar, obsessive-compulsive, or eating disorders; (4) moderate to severe traumatic brain injury or other neurocognitive disorder; (5) severe or unstable medical conditions, (6) magnetic resonance imaging (MRI) contraindications, such as metal or metallic devices in the body; (7) noncorrectable vision or hearing problems; and (8) current use of psychotropic medications that could affect brain function (e.g., anxiolytics, antipsychotics, or mood stabilizers). Participants reporting current use of antidepressants (SSRIs) are included as long as the dose has been stable for 6 weeks prior to enrollment. Inclusion/exclusion criteria are meant to decrease potential confounders while also supporting generalizability of results to GAD patient populations in the community.

### Intervention

Both BA and EXP treatments consist of manualized, ten-session interventions and are delivered in a group format for 90 min per week. For each group, participants are provided a binder to accompany the intervention, including outlines of each session, basic descriptions of concepts, and “homework” worksheets. Brief descriptions of each intervention are provided below and in Table 1. Descriptions of treatment compliance assessments and strategies are provided in Additional file 1.

**Table 1:** Primary goals for of each session involved in behavioral activation and exposure-based therapy protocols

Session	Exposure therapy content	Behavioral activation content
Pregroup	Brief pregroup individual check-in session: complete or review the Tulsa Life chart, provide a summary of the format and focus of exposure therapy	Brief pregroup individual check-in session: complete or review the Tulsa Life chart, provide a summary of the format and focus of behavioral activation
1	Session content: provide a brief overview of anxiety and depression and the rationale for exposure-based therapy Homework: initiate self-monitoring	Session content: provide a brief overview of anxiety and depression and the rationale behavioral activation Homework: Initiate self-monitoring
2	Session content: homework review, further discuss the role of conditioning and avoidance behavior in anxiety, begin building an exposure list Homework: continue self-monitoring and adding to exposure list	Session content: homework review, introduce concept of working from the “outside in” and discuss values as a way of identifying potential behaviors that may improve mood Homework: continue self-monitoring complete values worksheet
3	Session content: homework review, further develop exposure hierarchies, design and implement initial imaginal exposures Homework: continue self-monitoring, conduct repeated imaginal exposure exercises	Session content: homework review, discuss goal-dependent versus mood-dependent behavior and “acting as if,” discuss initial activity scheduling Homework: continue self-monitoring, engage in planned value-/goal-driven activities
4	Session content: homework review, conduct repeated imaginal exposures, discuss strategies for optimizing exposure exercises, design and complete an *in vivo* exposure exercise Homework: continue self-monitoring, conduct repeated imaginal and *in vivo* exposure exercises	Session content: homework review, introduce concept of function versus form and the tracking of antecedent, behavior, and consequences to examine function of behavior Homework: continue self-monitoring, use “ABC” sheets to examine function of behaviors, engage in planned value-/goal-driven activities
5	Session content: homework review, conduct repeated imaginal and/or *in vivo* exposure exercises, discuss strategies for optimizing exposure exercises. Homework: continue self-monitoring, conduct repeated imaginal and *in vivo* exposure exercises	Session content: homework review, introduce concept of avoidance as it relates to mood, values, and value-/goal-motivated behaviors. Homework: continue self-monitoring, use “ABC” or “TRAP/TRAC” worksheets to examine function of avoidance behavior, engage in planned value-/goal- driven activities
6	Session content: homework review, conduct repeated imaginal and/or *in vivo* exposure exercises, discuss strategies for optimizing exposure exercises Homework: continue self-monitoring, conduct repeated imaginal and *in vivo* exposure exercises	Session content: homework review, discuss how to integrate behavioral activation into their lives using “ACTION” Homework: continue self-monitoring, use “ABC” or “TRAP/TRAC” worksheets to examine function of behavior, engage in planned value-/goal-driven activities
7	Session content: homework review, conduct repeated imaginal and/or *in vivo* exposure exercises, discuss strategies for optimizing exposure exercises Homework: continue self-monitoring, conduct repeated imaginal and *in vivo* exposure exercises	Session content: homework review, discuss strategies to reduce the behavior of rumination, including “cueing action” and “attending to experiences” Homework: continue self-monitoring, use “ABC” or “TRAP/TRAC” worksheets to examine function of behavior, engage in planned value-/goal-driven activities, including those targeting rumination
8	Session content: homework review, conduct repeated imaginal and/or *in vivo* exposure exercises, discuss strategies for optimizing exposure exercises Homework: continue self-monitoring, conduct repeated imaginal and *in vivo* exposure exercises	Session content: homework review, discuss using behavioral activation to build the meaningful life that you want Homework: continue self-monitoring, use “ABC” or “TRAP/TRAC” worksheets to examine function of behavior, engage in planned value-/goal-driven activities
9	Session content: homework review, conduct repeated imaginal and/or *in vivo* exposure exercises, discuss strategies for optimizing exposure exercises Homework: continue self-monitoring, conduct repeated imaginal and *in vivo* exposure exercises	Session content: homework review, discuss troubleshooting techniques to counteract activation barriers Homework: continue self-monitoring, use “ABC” or “TRAP/TRAC” worksheets to examine function of behavior, engage in planned value-/goal-driven activities
10	Session content: homework review, conduct repeated imaginal and/or *in vivo* exposure exercises, repeat initial exposure exercises from session 3 to observe progress, discuss relapse prevention strategies Homework: conduct repeated imaginal and *in vivo* exposure exercises planned for the next 2–3 weeks and ongoing	Session content: homework review, reflect on and review previously learned techniques, discuss relapse prevention strategies Homework: continue to engage in self-monitoring as needed, engage in value-/goal-driven activities planned for the next 2–3 weeks and ongoing
Post	Brief postgroup individual wrapup session: update Tulsa Life chart, discuss observed trajectory of self-reported behaviors and symptoms through treatment, discuss treatment referrals as needed	Brief postgroup individual wrapup session: update Tulsa Life chart, discuss observed trajectory of self-reported behaviors and symptoms through treatment, discuss treatment referrals as needed

*Abbreviations: ABC* antecedent, behavior, and consequence, *TRAP* trigger, response, avoidance pattern, *TRAC* trigger, response, alternative coping, *ACTION* assess behavior/mood, choose alternate responses, try out alternate responses, integrate these alternatives, observe results, now evaluate

#### BA

BA is a recognized efficacious treatment for MDD [36] and is based on the premise that negative or stressful life events can reduce one’s ability to experience reward or reinforcement (e.g., reduced social support). Depression develops and is maintained when individuals respond in ways that create additional deficits in reward or reinforcement (e.g., further isolation). The goal of BA is to identify alternative behaviors to increase in a way that increases opportunities for reward or reinforcement, particularly through naturally reinforcing behaviors (e.g., those related to one’s values). A ten-session, structured, group-based BA manual was developed by coauthors RLA and CM (with edits and revisions provided by AC), informed by previously published BA treatment guides [29] and modified to focus on negative mood more generally rather than solely on depression.

#### EXP

EXP is a recognized efficacious strategy for the treatment of anxiety disorders. EXP is based on the premise that anxiety arises from a perceived threat associated with discrete cues or contexts, whether from direct or indirect/vicarious experience or informational transmission of perceived threat. Anxiety is thought to be maintained by avoidance behavior, preventing corrective learning. EXP guides individuals to decrease avoidance and experience anxiety-provoking situations or cues in a safe environment, allowing for inhibitory learning or habituation. The ten-session, structured, group-based EXP manual was based on a previous group-based anxiety treatment manual [37] developed by MGC, modified further by MGC and RLA (with edits and revisions provided by KWT and AC), to focus on exposure strategies only (without cognitive restructuring) and inhibitory learning rather than habituation only [38].

#### Therapist training and treatment fidelity

Each EXP and BA group intervention is delivered by two cotherapists: a licensed doctoral- or master’s-level clinician with either another licensed clinician or a therapist in training (i.e., clinical psychology postdoctoral fellow or graduate student). Each therapist completes in-person or online workshops (e.g., Behavioral Tech, LLC, https://behavioraltech.org; Centre for Research on Eating Disorders at Oxford, https://credo-oxford.com), reads articles and manuals related to each treatment [29, 38], and watches videos of previous therapy sessions. Each therapy session is video and audio recorded, and at least 20% of sessions will be randomly selected for fidelity ratings. Skill acquisition and fidelity are assessed using the Quality of Behavioral Activation Scale for BA (Dimidjian, Hubley, Martell, Herman-Dunn, and Dobson, 2012, unpublished measure) and a fidelity form created for the EXP treatment by RLA in consultation with MGC and KWT. Fidelity ratings will be provided by experts in each therapy (KWT and CM) or their trainees. Each therapist attends weekly consultation and supervision with the PI and/or consultants.

#### Data collection

All interview-based assessments (e.g., MINI) are administered by experienced, blinded examiners trained to high levels of interrater reliability (kappa > 0.80). Self-report data are collected electronically using Research Electronic Data Capture (REDCap) [39]. Study consent records are stored in a locked records room at LIBR. Study data records and blood/urine/biological samples are assigned code numbers and are not individually identifiable. REDCap servers are housed in a local data center at LIBR, and all web-based information transmission is encrypted.

### Measures

Self-report, behavioral, and neuroimaging measures included in the protocol are listed in Table 2 (refer to Fig. 2 for timing of measures). Below are descriptions of the behavioral and neuroimaging tasks serving as primary predictors of interest. The remaining tasks are described in Additional file 1.

**Table 2:** Diagnostic, demographic, self-report, behavioral, and neuroimaging assessments

Diagnostic and demographic assessment	
Diagnosis	MINI 6.0 or 7.0 [40]
History	Assessment of medical and medication history
Treatment completion	Intent to complete treatment form
History	Tulsa Life chart interview (see Additional file 1)
Standard self-report scales	
Negative valence	Symptoms of Depression Questionnaire (SDQ) [41]
Negative valence	Overall Anxiety Severity and Impairment Scale (OASIS) [35]
Negative valence	State-Trait Anxiety Inventory (STAI) [42]
Negative valence	Anxiety Sensitive Index (ASI-3) [43]
Negative valence	Generalized Anxiety Disorder–7 item (GAD-7) [44]
Negative valence	Intolerance of Uncertainty Scale (IUS) [45]
Negative valence	Penn State Worry Questionnaire [33]
Negative valence	Liebowitz Social Anxiety Scale (LSAS) [46]
Negative valence	Panic Disorder Severity Scale (PDSS) [47]
Negative valence	Beck Depression Inventory-II (BDI-II) [32]
Negative valence	Patient Health Questionnaire-9 [48]
Negative valence	Behavioral Activation for Depression Scale (BADS) [49]
Substance use	Customary Drinking and Drug Use Record (CDDR) [50]
Trauma	Traumatic Events Questionnaire (TEQ) [51]
Trauma	Child Trauma Questionnaire (CTQ) [52]
Positive/negative valence	Positive and Negative Affect Schedule (PANAS) [53]
Positive/negative valence	Behavioral Inhibition System/Behavioral Approach Scale (BIS/BAS) [54]
Comorbid anxiety symptoms	Padua Inventory of Obsessive-Compulsive Symptoms (PI) [55]
Personality	Big Five Inventory (BFI) [56]
Arousal/interoception	Multidimensional Assessment of Interoceptive Awareness (MAIA) [57]
Sleep	Pittsburgh Sleep Quality Index (PSQI) [58]
Physical activity	International Physical Activity Questionnaire (IPAQ) [59]
Disability	Sheehan Disability Scale (SDS) [30]
Therapy expectancies	Credibility/Expectancy Questionnaire (CEQ) [60]
Therapy compliance	Homework Rating Scale (HRS) [61]
Therapy process	Working Alliance Inventory (WAI) [62]
Therapy dropout	Withdrawn Questionnaire (see Additional file 1)
Pre/post neuroimaging	Karolinska Sleepiness Scale: prescan (KSS) [63]
Pre/post neuroimaging	Positive and Negative Affect Schedule: prescan (PANAS) [53]
NIH PROMIS® [64] and Toolbox® [65] measures	
Negative valence	PROMIS Anxiety
Negative valence	PROMIS Depression
Sleep	PROMIS Sleep Disturbance
Sleep	PROMIS Sleep-Related Impairment
Social	PROMIS Emotional Support
Social	PROMIS Information Support
Social	PROMIS Instrumental Support
Social	PROMIS Social Isolation
Sex	PROMIS Global Satisfaction with Sex Life
Sex	PROMIS Interest in Sex Activity
Nicotine	Nicotine dependence
Negative affect-anger	NIH Toolbox Anger-Affect Survey
Negative affect-anger	NIH Toolbox Anger-Hostility Survey
Negative affect-anger	NIH Toolbox Anger-Physical Aggression Survey
Negative affect-fear	NIH Toolbox Fear-Affect Survey
Negative affect-fear	NIH Toolbox Fear-Somatic Arousal Survey
Psychological well-being	NIH Toolbox General Life Satisfaction Survey
Psychological well-being	NIH Toolbox Meaning and Purpose Survey
Psychological well-being	NIH Toolbox Positive Affect Survey
Social	NIH Toolbox Friendship Survey
Social	NIH Toolbox Loneliness Survey
Stress and self-efficacy	NIH Toolbox Perceived Stress Survey
Stress and self-efficacy	NIH Toolbox Self-Efficacy Survey
Behavioral and neuroimaging tasks (see Additional file 1 for further description)	
Approach/avoidance	Implicit approach/avoidance task
Approach/avoidance	Attentional bias/dot probe task [66]
Approach/avoidance	Signal detection reinforcement task
Approach/avoidance	Human behavioral pattern monitor (hBPM)
Estimated IQ	Wide Range Achievement Test (WRAT) [67]
Neuropsychological	Delis-Kaplan Executive Function System (DKEFS) color-word test [68]
Neuropsychological	DKEFS verbal fluency [68]
Neuropsychological	Wechsler Adult Intelligence Scale (WAIS-IV) digit span [69]
Neuropsychological	WAIS-IV digit symbol coding [69]
Neuropsychological	Finger Tapping Test
Neuropsychological	California Verbal Learning Test (CVLT) [70]
Neuroimaging	MRI anatomical scan (T1-weighted)
Neuroimaging	fMRI resting state with eyes open
Neuroimaging	Approach-avoidance conflict (AAC) task
Neuroimaging	Emotional faces task (EFT)
Neuroimaging	Monetary incentive delay (MID) task

#### AAT

The AAT assesses behavioral avoidance tendencies [71]. Participants are shown a picture of an emotional face (happy, angry, or neutral) framed by a blue or yellow border and instructed to pull a joystick (approach) when the border is one color and to push it away (avoid) when it is the other (counterbalanced). The picture zooms out and in accordingly. Mean response latency for push is subtracted from pull (e.g., angry pull − angry push) to obtain an avoidance bias score.

#### AAC task

**Fig. 3:**
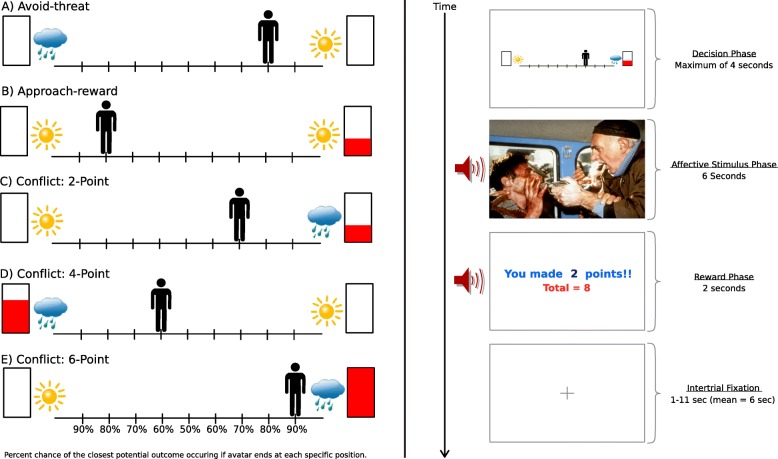
Approach-avoidance conflict (AAC) task. This figure displays (1) example decision screens displayed during the task for each of the five conditions: avoid-threat, approach-reward, and conflict with 2, 4, or 6 cents offered and (2) the sequence of screens presented for each AAC trial, including a decision phase followed by presentation of the affective image and sound pair (e.g., from International Affective Picture System and International Affective Digitized Sounds system [72, 73]), display of the number of cents received, and then a fixation cross until the next trial begins

The AAC task probes decision-making processes during approach-avoidance conflict [23, 24] (Fig. 3). On each trial, the subject decides between two outcomes, which are represented on each side of a runway. A cloud indicates that a negative affective image/sound pair will be shown, and a sun indicates that a positive image/sound pair will be shown (e.g., from International Affective Picture System and International Affective Digitized Sounds System [72, 73]). The amount of red in a rectangle indicates the amount of money awarded for each option (2, 4, or 6 cents). For conflict trials, negative stimuli are paired with rewards. Thus, the same behavior leads to both affective punishment and reward. For nonconflict “approach” trials, both outcomes include positive affective stimuli, but only one offers a reward. For “avoid” trials, neither outcome offers a reward, but one involves a negative affective image. For each trial, the subject moves the avatar, knowing that the probability of each outcome (10–90%) depends on their end position. Behavioral variables include approach behavior (end avatar position) and response time (RT) for initial button press.

During behavioral tasks, the BioPac MP150 system and Acq*Knowledge* software (BIOPAC Systems, Inc., Goleta, CA, USA) are used to collect galvanic skin conductance, heart rate (electrocardiogram), and respiration rate (respiration transducer). MRI data are collected on a GE MR750 3.0T MRI scanner (GE Healthcare Life Sciences, Chicago, IL, USA). Analysis of Functional NeuroImages [74] is used for processing of MRI data. Electroencephalography (EEG) is simultaneously performed during MRI scanning using a 31-electrode cap attached to an MRI-compatible BrainAmp MR Plus amplifier (Brain Products GmbH, Gilching, Germany). Blood samples for plasma, serum, and peripheral blood mononuclear cells are collected at baseline and posttreatment to quantify biomarkers for future exploratory analyses. See Additional file 1 for further description of MRI, EEG, and blood biomarker methods.

### Analysis procedures

The characteristics of all measures will be examined for missing data and deviation from normality prior to subsequent analyses. Baseline demographic characteristics and attrition data will be contrasted between treatment groups, and analyses will be adjusted to account for potential confounders. For the first aim, we will test the hypotheses that approach and conflict arbitration behavior, and neural responses will explain significant variance in baseline symptoms above and beyond avoidance-related behavior and neural responses. Approach and avoidance behavior are defined by AAT bias scores; conflict arbitration is defined by RT during AAC conflict trials. For brain responses, we will focus on the AAC task and extracted percentage signal change from the following *a priori* regions of interest:

1. *Approach*: left caudate (reward versus no-reward outcome)
2. *Avoidance*: right amygdala (negative versus positive affective outcome)
3. *Conflict*: right dlPFC (conflict versus nonconflict decisions)

We will use Huber robust regression with baseline GAD-7 as the dependent variable (DV) and approach, avoidance, and conflict measures as independent variables (IVs).

For the second aim, we will test the hypotheses that approach-related and conflict arbitration behavior and neural responses will predict treatment response above and beyond avoidance-related behavior and neural responses. We will use linear mixed models (LMEs) with random subject-level intercepts and slopes, GAD-7 scores across the ten sessions as DVs; baseline GAD-7 as a covariate; and approach, avoidance, and conflict measures as IVs. The main effect of intervention type and its interaction with IVs will be included to determine treatment main effects and moderating effects. We will determine the best set of IVs using the Lasso method [75], and we will use functional linear models [76] to model on-parametric symptom trajectories as needed.

For the third aim, we will test the hypothesis that the degree to which conflict arbitration abilities increase with treatment will positively relate to functional improvement from pre- to posttreatment. We will use LMEs to test main and interaction effects between intervention type and change in AAC conflict arbitration in predicting trajectories of GAD-7 scores over the ten sessions. We will employ the asymmetric distribution of product of coefficients test (versus Baron and Kenny methods) due to the greater power and more appropriate type I error rate it affords [77].

In addition, we are collecting data from other measures for exploratory analyses. For such analyses, we will explore (1) data reduction methods to derive multilevel factors associated with approach, avoidance, and conflict arbitration processes; and (2) use of random forest machine learning, which is particularly appropriate with a large ratio of predictors to participants [78], to identify predictors of treatment outcome.

### Sample size and power analysis

Previous research suggests large effects for fMRI predictors (i.e., *r* = 0.60–0.75) and medium to large effects for behavioral predictors (*r* = 0.30–0.47) of intervention outcomes [79, 80]. For this study, we aim to recruit 100 participants, which with 20% attrition would allow for complete longitudinal data for 80 participants (i.e., ~ 40/intervention). LMEs will include all participants with *any* postbaseline assessments. Thus, we anticipate approximately 50 participants per intervention for aims 2/3. For relationships between individual predictors and DVs, we estimate having 80% power to detect medium to large effects (*r* = 0.27 for *N* = 100; *r* = 0.37 for *N* = 50; *α* = 0.05). In a model with three predictors (approach, avoidance, conflict arbitration), we also estimate having 80% power to detect medium to large effects (η^2^ = 0.11 for *N* = 100; η^2^ = 0.24 for *N* = 50).

### Design considerations

We considered an alternative design where we examined predictors of EXP response compared with an attentional control intervention. We instead decided to identify unique predictors of two theoretically divergent behavioral therapies because (1) the current protocol was not meant to test intervention efficacy compared with “placebo” (because efficacy has been established in previous research); (2) variability in outcomes would be greatest for efficacious, as opposed to placebo, interventions, thus enhancing statistical power for prediction; and (3) identifying unique predictors for two therapies would be most clinically meaningful. We also considered using more unified cognitive behavioral interventions [81]. However, interventions that simultaneously target multiple processes (e.g., both cognitive and behavioral strategies) would have made it more difficult to identify predictors relating to the specific therapeutic target. The goal here is to foster individualized, precision targeting of treatments rather than to apply more broadly targeted treatments to all patients.

### Ethics

Study approval was obtained from the Western Institutional Review Board (WIRB) (protocol 20151232) Additional file 3. Any protocol modifications will be made to records at ClinicalTrials.gov and communicated to study investigators, WIRB, and funding organizations as required.

#### Gender/minority/pediatric inclusion for research

Planned study enrollment is reflective of Tulsa County population demographics and is described further in Additional file 1. We will not exclude subjects based on sex, gender, race, or ethnicity. Children are not included, owing to our initial focus on understanding neurobehavioral predictors of exposure therapy for adults with GAD. The variability introduced by developmental changes could reduce sensitivity to detect hypothesized relationships.

#### Safety

The risk for adverse events is minimal. The study is a clinical trial but is not a phase III trial, involves only one site, is not blinded, and does not employ high-risk interventions or vulnerable populations. The interventions employed are known to be efficacious for the treatment of anxiety or depression. The PI (RLA), in collaboration with mentor and LIBR scientific director (MPP), are performing the monitoring function for the study. Any unanticipated adverse events will be reported immediately to the LIBR Human Protection Administrator and to the Western IRB. Any adverse events will be included in the annual IRB report.

Each week prior to session, participants complete questionnaires assessing symptom severity and suicidal ideation. As needed, a therapist meets with the participant individually to assess risk and provide referrals or identify emergency services. LIBR is situated on a campus with an inpatient psychiatric hospital (Laureate Psychiatric Clinic and Hospital), which has a 24-h clinical assessment department.

#### Dissemination

Results will be shared with the scientific and health professional community through presentation at national and international scientific meetings and publication in scientific journals. The full protocol and statistical code used for data analysis will be provided with resulting publications. To disseminate results to the general public, all final peer-reviewed manuscripts will be submitted to the PubMed Central digital archive in compliance with the NIH public access policy. The PI will maintain a local website where lay summaries of scientific results will be provided, with appropriate links to scientific presentations and publications. The PI will ensure that the summary of results information is submitted to ClinicalTrials.gov.

## Discussion

To enhance treatment effectiveness and efficiency for individuals with anxiety and depression, it will be beneficial to understand why many patients do not respond optimally to gold standard therapies and to be able to predict, before treatment begins which patients will respond to which treatments. The study detailed in this protocol article represents a first step toward these goals, using the RDoC framework [12] to probe multilevel predictors of EXP versus BA therapy for GAD. This work addresses the NIMH strategic plan by (1) integrating biological markers and behavioral indicators associated with GAD (strategy 1.3) and (2) using a multidimensional design to ascertain individual predictors of therapy response (strategy 3.1) that will (3) inform future research developing strategies for personalized mental health care (strategy 3.2).

Czajkowski et al. [82] presented the Obesity-Related Behavioral Intervention Trials (ORBIT) model as a strategy for the development of novel behavioral treatments. Although the ORBIT model was developed from a health psychology perspective, it is also relevant more broadly. These authors proposed that “the hypothesis that change in a behavioral risk factor could solve a clinical problem is one of the entry points for behavioral treatment development” [82]. Similarly, the identification of neural and behavioral risk factors for response or nonresponse to different psychosocial treatments could provide entry points for development of novel, personalized mental health treatment strategies. If specific behaviors (i.e., conflict arbitration difficulties, approach motivation) and/or neural networks (e.g., dlPFC, striatum) can be shown to predict therapy outcomes, research could then turn toward identifying strategies for modifying these factors. This could involve neuromodulation approaches (e.g., fMRI real-time neurofeedback, transcranial magnetic stimulation) [83], cognitive or behavioral strategies (e.g., cognitive control or attention bias training, cognitive rehabilitation strategies [84, 85]), or pharmacologic approaches (e.g., dopaminergic or *N*-methyl-d-aspartate-related drugs to target motivational or cognitive circuitry, respectively) [86]. This approach is in concert with NIMH’s more recent experimental therapeutic approach to clinical trials.

Strengths of the described protocol include the randomization of participants to two interventions, both of which have documented efficacy but target different and specific processes rooted in distinct neural circuitry. The study is strengthened by inclusion of multilevel assessments—self-report, behavioral, and neurobiological—to probe domains of positive and negative valence and cognitive control. In addition, the domains and interventions assessed are relevant transdiagnostically. Thus, if promising results are identified, future studies could use similar protocols to test whether findings can be generalized to other anxiety disorder and depressive disorder populations. In addition, the interventions are manualized and identical in regard to format, frequency, duration, and level of therapist training, and we use consultation with experts in each.

The study is not without limitations. Although our target sample size is larger than any published fMRI study predicting GAD treatment response, the sample size is underpowered to detect small effect sizes or for independent replications. Thus, results identified from the current study will require follow-up replication. Also, the trial is being conducted at only one site, so generalizability across sites would need to be determined in future research. The delivery of the intervention in a group format allows for greater control and balance regarding which therapists are providing the treatment, and it increases cost and time efficiency of the trial. However, this may limit the generalizability of findings to individual therapy.

This protocol provides a framework for how studies may be designed to move the field toward neuroscience-informed and personalized psychosocial treatments. The results of the trial will have implications for approach-avoidance processing in GAD; relationships between levels of analysis (i.e., behavioral, neural); and, most important, predictors of behavioral therapy outcome. The results also have the potential to inform a line of research aimed at optimizing psychosocial treatment for anxiety and depressive disorders from a holistic, neuroscience, and behaviorally informed perspective and to move us closer to truly personalized precision approaches to psychiatric treatment.

## Trial status

Study approval was obtained from the Western Institutional Review Board (WIRB; protocol 20151232). Recruitment began on June 7, 2016, and the approximate date when recruitment will be completed is April 1, 2021. The study was retrospectively registered within 21 days of first participant enrollment in accordance with FDAAA 801 (ClinicalTrials.gov identifier NCT02807480; registration date June 21, 2016).

## Supplementary information

**Additional file 1.** Supplementary material: Additional information on methods and analysis of the interventions, assessments (behavioral and neuroimaging), blood biomarker storage, and plans for enrollment.

**Additional file 2.** SPIRIT checklist: Information regarding the recommended items for a clinical trial protocol paper.

**Additional file 3.** Approval letter from Western Institutional Review Board.

**Additional file 4.** K23 Notice of Award: Letter from the National Institute of Mental Health informing the principal investigator of the award granted for this protocol.

**Additional file 5.** Study consent form: The informed consent document approved by the Western Institutional Review Board.

## Data Availability

The datasets analyzed during the current study are available from the corresponding author on reasonable request.

## Abbreviations

AAC: Approach-avoidance conflict
AAT: Approach-Avoidance Task
ABC: Antecedent, behavior, and consequence
ACTION: Assess behavior/mood, choose alternate responses, try out alternate responses, integrate these alternatives, observe results, now evaluate
BA: Behavioral activation
BADS-SF: Behavioral Activation for Depression Scale–Short Form
BDI-II: Beck Depression Inventory II
BDI-II SI: Beck Depression Inventory suicidal ideation item
CBT: Cognitive behavioral therapy
CEQ: Credibility/Expectancy Questionnaire
dlPFC: Dorsolateral prefrontal cortex
DSM-5: *Diagnostic and Statistical Manual of Mental Disorders, Fifth Edition*
DV: Dependent variable
EEG: Electroencephalography
EXP: Exposure therapy
fMRI: Functional magnetic resonance imaging
GAD: Generalized anxiety disorder
GAD-7: Generalized Anxiety Disorder 7-item scale
HRS: Homework Rating Scale
IV: Independent variable
LIBR: Laureate Institute for Brain Research
LME: Linear mixed models
LSAS: Liebowitz Social Anxiety Scale
MDD: Major depressive disorder
MINI: Mini International Neuropsychiatric Interview
MRI: Magnetic resonance imaging
NIH: National Institutes of Health
NIMH: National Institute of Mental Health
OASIS: Overall Anxiety Severity and Impairment Scale
ORBIT: Obesity-Related Behavioral Intervention Trials
PDSS: Panic Disorder Severity Scale
PFC: Prefrontal cortex
PROMIS Anx & Dep: Patient-Reported Outcomes Measurement Information System anxiety and depression scales
PSC: Percentage signal change
PSWQ: Penn State Worry Questionnaire
RDoC: Research Domain Criteria
REDCap: Research Electronic Data Capture
RT: Response time
SDS: Sheehan Disability Scale
SPIRIT: Standard Protocol Items: Recommendations for Interventional Trials
SSRI: Selective serotonin reuptake inhibitor
TRAC: trigger, response, alternative coping
TRAP: trigger, response, avoidance pattern
Tx: treatment
WAI: Working Alliance Inventory
WIRB: Western Institutional Review Board
