# Supplementary material for: Protocol for a randomized controlled trial examining multilevel prediction of response to behavioral activation and exposure-based therapy for generalized anxiety disorder
Source: Trials. 2020 Jan 6;21:17. doi: 10.1186/s13063-019-3802-9 (PMC6943897; doi:10.1186/s13063-019-3802-9)
Supplement: Supplementary file 1 — Additional file 1. Supplementary material: Additional information on methods and analysis of the interventions, assessments (behavioral and neuroimaging), blood biomarker storage, and plans for enrollment. [file 13063_2019_3802_MOESM1_ESM.docx]

**Protocol for a Randomized Controlled Trial Examining Multi-level Prediction**

**of Response to Behavioral Activation and Exposure-based Therapy for Generalized Anxiety Disorder**

**SUPPLEMENTARY MATERIAL**

Santiago, J.^1^, Akeman, E.^1^, Kirlic, N.^1^, Clausen, A.N.^5,6^, Cosgrove, K. T.^1,3^, McDermott, T. J.^1,3^, Mathis, B.^1^, Paulus, M.^1,2^, Craske, M. ^7^, Abelson, J.^8^, Martell, C.^9^, Wolitzky-Taylor, K.^7^, Bodurka, J. ^1^, Thompson, W.^10^, & Aupperle, R. L.^1,2^

^1^Laureate Institute for Brain Research, Tulsa, OK, USA

^2^School of Community Medicine, University of Tulsa, Tulsa, OK, USA

^3^Department of Psychology, University of Tulsa, Tulsa, OK, USA

^4^Health Services & Outcomes Research, Children’s Mercy Hospital, Kansas City, MO, USA

^5^VA Mid-Atlantic Mental Illness Research, Education and Clinical Center, Durham, NC, USA

^6^Duke University Brain Imaging and Analysis Center, Durham, NC, USA

^7^Psychology, Psychiatry and Biobehavioral Sciences, University of California – Los Angeles, Los Angeles, CA, USA

^8^Department of Psychiatry, University of Michigan, Ann Arbor, MI, USA

^9^Department of Psychology and Brain Sciences, University of Massachusetts - Amherst, Amherst, MA, USA

^10^Family Medicine and Public Health, University of California – San Diego, San Diego, CA, USA

**Short Title:** Prediction of Treatment Response for Generalized Anxiety Disorder

***Corresponding Author**

Robin Aupperle, PhD

Laureate Institute for Brain Research

6655 S. Yale Ave., Tulsa, OK 74136, USA

Tel: 918-502-5744; Fax: 918-502-5135

E-mail: raupperle@laureateinstitute.org

**Study sponsor**

Laureate Institute for Brain Research

6655 S. Yale Ave., Tulsa, OK

Phone: 918-502-5100; Fax: 918-502-5135

E-mail: info@laureateinstitute.org

Keywords: generalized anxiety disorder, depression, behavioral activation, exposure therapy, cognitive-behavioral therapy, functional magnetic resonance imaging

**SUPPLEMENTAL METHODS AND ANALYSES**

**Intervention**

*Treatment compliance:* At the pre-treatment individual therapy introduction session, all participants are asked how likely they are to be able to attend all ten sessions (on a 1-10 scale, with 10 being extremely likely) and any obstacles that might interfere with session attendance is discussed. Group sessions are conducted in the evening (e.g., after 5pm) to reduce conflicts with work or other responsibilities. Participants are sent text or voicemail reminders prior to each session. If a subject does not attend a group session, a therapist contacts the subject via phone to check in briefly and summarize the content of the session and assigned homework. Participants who do not attend sessions are sent a link via email to complete their weekly surveys. The Homework Rating Scale (HRS(1)) is administered weekly during the interventions to assess for compliance with between-session assignments. Treatment may be discontinued for a trial participant due to a worsening of mental health symptoms that require immediate attention or treatment, participant request, or if they miss more than two of the ten group therapy sessions. If a subject withdraws from the study, they are asked to complete a questionnaire asking for reasons they withdrew(2, 3) or could not complete treatment (questionnaire developed by RLA, based on previous research in other mental health populations(2, 3)), and primary symptom outcome measures at the time of withdrawal. Regardless of treatment completion, all participants are asked to complete 3 and 6 month follow-up symptom measures.

**Interview-based and Self-report Assessments**

Descriptions of self-report assessments created or modified for this protocol are described below.

*Tulsa Life Chart (TLC):* Development of the TLC was informed by the general approach of the NIMH Life-Chart (4, 5), and by considering transdiagnostic psychosocial factors thought to contribute to mental health. The TLC is conducted as a structured interview by therapists and takes approximately 2.5 hours to administer. Training in TLC administration is supervised by a licensed clinical psychologist (RLA). The TLC interview assesses birth date, location, and birth complications, and then queries specific components from each of the following epochs: (i) birth to elementary school or age 5, (ii) elementary school or ages 5-10, (iii) middle school or ages 11-14, (iv) high school or ages 15-18, (v) young adult or ages 18-25, (vi-viii) and for every 10 years thereafter to the age of the participant (i.e., 25-35, 35-45, 45-55). For each epoch, participants provide an average mood rating (1-10, with 1 being the worst they have ever felt and 10 being the best they have ever felt) as well as the frequency/number, start/end dates, and brief descriptions for each of the following: (i) places lived (residences), (ii) schools attended, (iii) leisure activities (hobbies), (iv) employments (jobs), (v) people they felt close to (people), (vi) exposure or experiences with substances, (vii) mental health treatments, (viii) changes in family structure, e.g., birth of a child, marriages, divorces, and so forth (change events), (ix) negative (bad), (x) positive (good) events, and (xi) any “other” events the felt were important. Participants are asked to rate their mood at the time of each negative, positive, change, and other event, on the same 1-10 scale.

*Withdrawn Questionnaire:* The Withdrawn Questionnaire was developed based on previous research in other populations, to address potential reasons for termination of treatment, including therapy expectations (e.g., “The therapy sessions were too stressful”, “I do not believe therapy is useful for my symptoms”), therapy structure (e.g., “The therapy sessions were too long”, “I would have preferred individual rather than group therapy”), symptom severity (e.g., “My symptoms are no longer bothering me”, “My symptoms were so severe that it interfered with me coming to sessions”), study or staff variables (e.g., “I did not trust the therapist”, “I did not like/get along with the therapist”), external factors (e.g., “therapy times were inconvenient or conflicted with my schedule”, “I had childcare problems that kept me from coming in”). There is also a field for participants to fill in additional reasons.

**Behavioral Assessments**

Descriptions of each behavioral task are included below (except for the Approach Avoidance Test [AAT], which is described in the main text). During each of the behavioral tasks, the BioPac MP150 system and Acknowledge software (BIOPAC instrumentation; Lehigh, Pennsylvania) are used to collect galvanic skin conductance, heart rate (electrocardiogram), and respiration rate (respiration transducer). In addition, a video camera records each subject during the behavioral tasks in order to permit automatic, objective analysis of facial expressions for exploratory data analysis.

*Modified Probe Detection Task (MPDT)(6):* This task assesses attentional bias for positive and negative information. Each trial consists of brief presentation of a cue at a specific location on the screen (a small horizontal or vertical line), follow by a pair of images (one representational, one non-representational), and then a target (another horizontal or vertical line). The participant responds indicating whether the target is the same or different from the cue. Representational stimuli comprise international affective picture system (IAPS) images taken from positive, negative, or neutral valence sets. Non-representational images will consist of abstract art. Participants are presented with a total of 192 trials: 64 from each of positive, negative, and neutral images. The following traits are balanced across trials: representational image location, cue location, cue orientation, target location, target orientation, image duration (500 or 1000ms). Differences in reaction time for targets appearing congruent or noncongruent with affective image location are used to calculate positive and negative engagement and disengagement biases.

*Signal Detection Reinforcement Task (SDRT)(7):* SDRT assesses behavior modulation as a function of prior reinforcements. For each *SDRT* trial, a cartoon face (without a mouth) is displayed. A short or long mouth is shown for 100 ms (pseudo-randomized and counterbalanced). Participants identify by button press the type of mouth presented. One mouth type is associated with three times more positive feedback (monetary value) than the other. The task difficulty is calibrated such that out of 300 total trials the subject will be awarded for 120. For the entire task, participants are awarded ~$6. Severity of depression has been shown to relate to deficiencies in developing response biases for rewarded trials on this task. The SDRT lasts 10 minutes and will be conducted both at baseline and post-treatment.

*Human Behavioral Pattern Monitoring (HBPM) Paradigm:* This paradigm is based on previous work with other psychiatric populations(8) and assesses naturalistic exploratory approach and avoidance behavior by recording participants’ behavior when in a naturalistic setting (design to appear like a “child testing room”) filled with objects that invite exploration (toys, such as a ball, mask, puzzle). This task is meant to mimic paradigms used in animal studies to quantify approach-avoidance behavior (e.g., open field tests such as the BPM) and will provide a more “real-world” task to complement the computer-based tasks. Participants will be fitted with an Equivital Life Monitoring Sensory system(9), an ambulatory, multi-sensor, continuous monitoring vest for collecting, analyzing, and reporting health data, including electrical activity of the myocardium via a 3-lead electrocardiogram and activity/posture via a two-axis accelerometer.

**Neuroimaging assessments**

Descriptions of each neuroimaging task are included below (except for the Approach Avoidance Conflict task [AAC], which is described in the main text).

*Monetary Incentive Delay Task (MID)(10-12)*: The MID is among the most robust methodologies in eliciting striatal activation associated with processing and anticipation of reward. On each trial, participants are given a cue for potential reward (circle), loss (square), or no reward/loss (circle/ square). Horizontal lines in the cue indicate three reward/loss levels. To receive reward or avoid loss, participants quickly press a button after seeing a target (white square). Task difficulty is adjusted to reflect each subject’s skill level on the task using pre-scan reaction time assessment so that each subject will succeed on ~66% of trials and earns $0-55. Regressors of interest for analysis of neuroimaging data collected during the MID will include anticipation and processing of gain, non-gain, loss, and non-loss.

*Emotional Faces Task (EFT)(13)*: The visual presentation of emotional face stimuli is a robust methodology for eliciting amygdala activation and probing threat or emotion reactivity. Participants will be presented with a series of shapes or emotional faces(14) (angry, fearful, or happy) and will be instructed to choose between two options to match a target stimuli based on either form (skew direction) or emotion, respectively. For each trial, there will be a target shape/face and two options for matching. Regressors of interest for analysis of neuroimaging data collected during the EFT will include angry, fearful, happy and shape trials.

*MRI Processing:* Analysis of Functional NeuroImages (AFNI)(15) will be used for slice-time correction, volume registration, anatomical alignment, Talairach normalization, and full-width half-maximum smoothing to allow for calculation of percent signal change (PSC) associated with the regressors of interest for each task. Motion (roll, pitch, yaw) and linear, quadratic, and cubic trends will be entered as regressors of no interest for all paradigms.

*Electroencephalogram (EEG) Collection and Processing:* During fMRI, we will simultaneously record EEG using a 31-electrode cap attached to an MRI-compatible BrainAmp MR Plus amplifier. Ag/AgCl electrodes are cap-mounted using the standard 10-5 system, referenced to FCz, with an AFz ground electrode. Electrode impedance will be maintained <0 KΩ. A 5000 Hz sampling frequency will be used (analog filter of 0.016-250 Hz; 0.1 µV resolution). Besides independent EEG measures of brain state, we will use EEG data to correct the effects of head movements in fMRI data on a slice-by-slice basis using the E-REMCOR technique(16). EEG data will be corrected for MRI artifacts with the template subtraction method(17). Data will be band-pass filtered (1 to 70 Hz), downsampled to 250 Hz, and re-referenced to the common average reference. Fast Fourier transformation will estimate δ(1–3 Hz), θ (4–7 Hz), α (8-13 Hz), and β(13–30 Hz) frequency band spectral power. Log-normalized F3, Fz, and F4 power densities will be averaged.

**Blood biomarker storage**

Participants have a non-fasting blood drawn by venipuncture by a trained phlebotomist for the biomarker panels. Blood draws are scheduled to occur the morning of one of the baseline and one of the post-treatment visits (prior to 11am). Resting blood pressure and heart rate are also assessed during these visits. Less than 150 mL of blood is collected per subject during each session (baseline and post-treatment), which is well within the safety limit of ~450 mL per blood draw. Blood samples for plasma, serum and peripheral blood mononuclear cells (PBMCs) will be transported to and processed at the University of Oklahoma Integrative Immunology Center (IIC) Laboratories. Plasma and serum samples will be stored in secure freezers at −80$^{\circ}$C. Freezers will be maintained in a specially equipped room with emergency backup power and an automated telephone alarm system that is programmed to call in case of failure. Additional aliquots of samples will be stored at −80$^{\circ}$C should repeat analyses be required at a later date. PBMCs will be stored in liquid nitrogen dewars with liquid level monitors and alarms in a secure room at the University of Oklahoma IIC Laboratories.

**Gender/minority/pediatric inclusion for research:**

Our planned enrollment for the study is reflective of the population demographics in Tulsa county and anxiety disorder populations in general(18). We expect to enroll approximately 62% women (38% men); approximately 12% Hispanic/Latino (88% not Hispanic/Latino); approximately 9% American Indian or Alaska Native, 3% Asian, 0% Native Hawaiian or other Pacific Islander, 11% Black or African American, 7% more than one race, and 70% White. The population is approximately equal in regards to sex. However, anxiety has been reported to be more prevalent for women than men, with both lifetime and 12-month prevalence ratings being approximately 1 male to every 1.7 females(18). Thus, values for our targeted/planned enrollment closely approximate race/ethnicity distributions from recent census statistics for the state of Oklahoma and Tulsa county and the reported sex distributions for anxiety disorders.

**Additional items from SPIRIT Checklist**

**SPIRIT Item #3. Current protocol version: 4; last modified June 24, 2019.** The following provides a history and summary of protocol revisions:

v. 4.0, June 24, 2019: The website used for delivery of the historical interview, referred to as the Life Chart, used during pre-treatment assessment was modified from REDCap (Research Electronic Data Capture) to a secure virtual machine hosted by the Laureate Institute for Brain Research, with resulting data stored behind a firewall through a password protected database on a secure server managed by LIBR.

v. 3.0, March 28, 2016: (1) The GAD-7 self-report scale was added to be administered at the weekly therapy sessions, (2) A treatment follow-up form was added to the three- and six-month time points to assess for what types of treatment or medical problems participants may have experienced since completing the study, (3) A radio advertisement and Facebook advertisement were submitted to use for participant recruitment.

v. 2.0, January 22, 2016: (1) Added the Withdrawn Questionnaire, to be administered to participants who withdraw from the study prior to completion of treatment, (2) Addition of the 3- and 6-month follow-up time points, (3) Reduced and modified the measures being administered at weekly treatment sessions to reduce the burden to participants.

v. 1.0, December 14, 2015: (1) Modify the amount of time listed in the consent forms concerning completion of protocol measures to more accurately reflect the time required, (2) adding an optional consent form concerning the use of therapy videos and audios for training of clinicians outside of the institute, (3) adding further details concerning the randomization of participants to the two interventions.

v. 0.0, June 30, 2015: Original protocol approved.

**SPIRIT Item #5d. Composition, roles, and responsibilities of the coordinating centre, steering committee, endpoint adjudication committee, data management team, and other individuals or groups overseeing the trial.**

The only site for this study is the Laureate Institute for Brain Research (LIBR), who is therefore considered the coordinating centre. The study team at LIBR will serve the purpose of steering committee and data management team, and will provide oversight of the trial in coordination with the institutional review board (IRB). There is no Stakeholder and Public Involvement Group (SPIG) for the current study. Below, we have summarized the role of the different members of the research team and the meeting frequency to support the ongoing management of the study.

Principal Investigator: The proposed project includes one study site, Laureate Institute for Brain Research (LIBR). The Principal Investigator, Robin Aupperle, PhD, will initiate, lead, and oversee the proposed project, ensuring that all study staff are well trained in study procedures, providing supervision and oversight of all assessment, neuroimaging, and clinical procedures involved in the proposed protocol. She will ensure that the research is conducted ethically and rigorously. Dr. Aupperle will also oversee the analysis of clinical and functional magnetic resonance imaging (fMRI) data collected and lead the interpretation of results and writing of research results for presentation and publication. Dr. Aupperle is Principal Investigator and Clinical Psychology Interventions and Training at LIBR and Assistant Professor within the School of Community Medicine at The University of Tulsa (TU). She is well-suited to this role due to her expertise in conducting fMRI research related to anxiety and trauma-related disorders, and for the purposes of delineating treatment mechanisms and predictors.

Co-Investigators and K23 Award Mentors: Additional investigators include Drs. Martin Paulus, Jerzy Bodurka, Henry Yeh, and Namik Kirlic. Study consultants include Drs. Michelle Craske, Jim Abelson, Kate Wolitzky-Taylor, and Christopher Martell. Their specific roles are described below. All Co-Investigators and consultants will participate in data analytic plan, interpretation of the results, and preparation of manuscripts. Martin Paulus, M.D. is a board certified psychiatrist with specific expertise in fMRI research, specifically as a tool for understanding neural substrates of anxiety, depression, and addition, and in predicting longitudinal outcomes in these populations. Dr. Paulus is also the Scientific Director and President of LIBR. He will assist Dr. Aupperle in overseeing the clinical and longitudinal aspects of the research and provide his expertise in fMRI analyses. He will also participate in data interpretation and preparing manuscripts for publication. Jerzy Bodurka, Ph.D. has been the Neuroimaging Core Director, Chief Technology Officer, and Associate Professor at LIBR since 2009. He is an MRI physicist, with expertise in the field of Blood Oxygenation Level Dependent (BOLD) functional Magnetic Resonance Imaging (fMRI) and simultaneous acquisition of fMRI and EEG. Dr. Bodurka will be involved in experimental design and fMRI parameter optimization, and will be responsible for maintaining the MRI research environment, and ensure that the quality of data collected during MRI is of the highest quality. He will provide input concerning the MRI and fMRI data analysis procedures and will contribute to the interpretation of findings and manuscript preparation. Michelle Craske, Ph.D. is Director of the Anxiety Disorders Research Center and clinical psychologist at the University of California, Los Angeles (UCLA). Her research focuses on the etiology and treatment mechanisms for anxiety and related conditions and has extensive experience with treatment manual development for anxiety and behavioral outcome research with transdiagnostic anxiety and depression. Dr. Craske developed the exposure-based therapy manual used in the current project. During the project, she will consult with Dr. Aupperle monthly, providing input concerning the study design, and ongoing management of the randomized clinical trial, including retention strategies. Dr. Craske will also give crucial input for the interpretation of findings and manuscript preparation to disseminate results. Jim Abelson, PhD is Professor and Director of the Anxiety Program at the University of Michigan. He has specific expertise in the psychoneuroendocrinology of stress, and the psychological modulation of HPA axis activity and its dysregulation in mood and anxiety disorders. For the current project, Dr. Abelson will offer expertise in multilevel research integrating neuroscientific and behavioral measures and the use of basic science to inform clinical studies. During the project, Dr. Abelson will consult with Dr. Aupperle, providing input concerning the study design and give crucial input for the interpretation of findings and manuscript preparation to disseminate results. Namik Kirlic, Ph.D. is an associate investigator at LIBR. He is a licensed clinical psychologists with expertise in the implementation of empirically-supported therapy interventions for anxiety, depression, and trauma-related disorders, as well as fMRI preprocessing and analyses. For the current project, he will work closely with Dr. Aupperle and Ms. Santiago in overseeing completion of therapy groups. Specifically, he will assist in the delivery and supervision of the therapy interventions and assist in overseeing data management, processing, and analyses. He will also provide input on data interpretation and manuscript preparation.

Consultants: Wes Thompson, Ph.D. is a biostatistician and Professor In Residence, Family Medicine and Public Health, at the University of California – San Diego. He has expertise in analyzing longitudinal datasets, with a specific focus on fMRI data analysis and prediction of longitudinal outcomes. Dr. Thompson will provide consultation through the study concerning the data analysis plan and will provide crucial input on data interpretation and manuscript preparation. Kate Wolitzky-Taylor is Associate Professor and clinical psychologist within the Department of Psychiatry and Behavioral Sciences at University of California, Los Angeles (UCLA). Dr. Wolitzky-Taylor has expertise in research understanding the nature of anxiety disorders, as well as the development and implementation of evidence-based treatments. Dr. Taylor will provide weekly consultation during the implementation of exposure-based group therapy, and provided fidelity ratings for 40% of these therapy sessions. Christopher Martell is Director of Psychological Services Center, Lecturer, and clinical psychologist at the University of Massachusetts, Amherst. Dr. Martell has expertise in the delivery of behavioral interventions, and behavioral activation in particular, within both community-based and research settings. Dr. Martell and Dr. Aupperle developed the group-based behavioral activation therapy manual used in the current project. Dr. Martell will also provide weekly consultation during the implementation of behavioral activation group therapy, and provided fidelity ratings for 40% of these therapy sessions.

Study and Clinical Team: The Research Coordinator, Elisabeth Akeman, BA has experience in the coordination of several longitudinal clinical outcome and neuroimaging studies. For the current project, she will assist Dr. Aupperle in study implementation. More specifically, Ms. Akeman will: (1) recruit and screen interested individuals; (2) schedule eligible participants for baseline assessment sessions; (3) obtain informed verbal and written consent from participants; (4) administer clinical, behavioral, and neuroimaging assessments at each session; (5) work closely with Ms. Santiago to coordinate the transition of participants into group therapy, (6) monitor planned versus achieve recruitment/retention goals; and (6) manage and back up data collected at each session. Research Clinician, Jessica Santiago, LPC is a licensed professional counselor and Research Clinician at LIBR. For the current project, Ms. Santiago will work closely with Dr. Aupperle to ensure that the therapy intervention are implemented with high fidelity. Ms. Santiago has completed extensive training in supportive therapy, behavioral activation, and exposure-based treatment approaches. This includes workshops conducted by Dr. Craske’s laboratory on exposure-based approaches and by Dr. Martell on behavioral activation. She will work closely with Dr. Aupperle and Dr. Kirlic, in overseeing completion of therapy groups. Specifically, she will schedule participants for therapy groups, organize all materials for the therapy sessions, deliver the therapy interventions, and be responsible for any between-session clinical consultation required with participants (e.g., for participants who miss sessions or have treatment-related concerns).

Recruitment & Assessment Team: The LIBR Assessment team is composed of multiple research assistants trained to employ recruitment strategies and conduct initial screening assessments to determine eligibility. The Assessment team will work closely with the PI and Study Team to achieve an adequate flow of participants according to the study timeline, the adequacy of recruitment resources, and ensure eligibility of incoming volunteers. The Assessment Team will work closely with the Study Team to ensure timely telephone and in-person screening of volunteers, obtaining informed consent, administering diagnostic interviews and symptom rating scales in accordance with the study protocol, and proper coding, entry, and backup of study data.

Neuroimaging Team: The LIBR Neuroimaging Team consists of four experienced MRI technologists, a computer programmer, and three research scientists (Vadim Zotev, PhD; Misaya Misaki, PhD; Aki Tsuchiyagaito, PhD) and is overseen by Dr. Bodurka. The LIBR Neuroimaging Team will coordinate scheduling of MRI scans, perform daily quality assurance procedures, backup of study data, and work closely with the Study Team to ensure implementation of the imaging protocol and behavioral tasks according to the study research plan. The computer scientist will assist the PI to program fMRI and EEG tasks according to the study design.

Meeting Schedule: On a weekly basis, Dr. Aupperle (PI) will meet with Dr. Kirlic and study coordinator, Ms. Akeman, and research clinician, Ms. Santiago, to discuss the implementation of interventions, troubleshoot any potential issues with participant recruitment and retention, data collection, data management and backup, and data preprocessing. In addition, Dr. Aupperle (PI), Ms. Santiago (research clinician), and Dr. Kirlic will consult weekly with consultants, Drs. Wolitzky-Taylor and Martell concerning the implementation of exposure-based therapy and behavioral activation.On a monthly basis, The PI will meet with Co-Investigators, Drs. Paulus, Bodurka, and Kirlic monthly and as-needed to discuss recruitment goals, data collection/backup issues, preprocessing of data, data analysis/interpretation, and/or dissemination of results during the study.

Additional Consultation: The PI will also consult regularly with Drs. Craske and Abelson through conference calls and in-person meetings at scientific conferences concerning overall study progress, participant follow-up retention, data interpretation, and/or dissemination of results during the study. The PI will consult with Dr. Thompson concerning the data analysis plan and interpretation of results.

**SPIRIT Item #17a: Who will be blinded after assignment to interventions (e.g., trial participants, care providers, outcome assessors, data analysts), and how:**

Participants are kept blinded to which treatment condition they will be assigned until after completion of all baseline assessment measures, including clinician interviews, self-report measures, computer-based behavioral assessment, and fMRI scanning. Individuals conducting subjective, clinician-based interviews are kept blinded throughout the study. As the primary outcome variables are self-report measures (GAD-7, Sheehan Disability Scale, PROMIS Depression and Anxiety), neither participants nor administrators of these assessments were blinded during weekly and post-treatment assessment. Data analysts are blinded to intervention condition assignment of participants through the preprocessing stages, until conducting final group-based analyses. The PI and clinicians are not blinded to intervention condition.

**SPIRIT Item #17b:** If blinded, circumstances under which unblinding is permissible, and procedure for revealing a participant’s allocated intervention during the trial.

Because the intervention being provided is a therapy, the clinicians delivering the intervention and the PI (who is supervising delivery of the interventions) are not blinded. Thus, in emergency situations or cases in which the individual must be referred to further care external to the study (e.g., due to worsening of symptoms) – the clinicians will already be aware of intervention condition in order to adequately respond to these situations. After baseline assessments, the participants are told which intervention they are randomized to complete and from then on out, are not blinded to treatment condition. The PI and clinicians are not blinded to intervention condition and thus, unblinding is not applicable.

**SPIRIT Item #21b: Description of any interim analyses and stopping guidelines, including who will have access to these interim results and make the final decision to terminate the trial.**

We did not have any formal stopping rules because we are utilizing two interventions of known efficacy for generalized anxiety disorder (GAD), rather than novel interventions with unknown efficacy and side effects. The main focus of the study is on what factors predict outcomes to these interventions. We therefore did not feel that such stopping rules and interim analysis was necessary in this case, and it was not required as part of the institutional review board (IRB) and National Institute of Health (NIH) grant review process. It is the PI’s responsibility to monitor any adverse events that occur during the intervention and report them to the IRB of record (Western Institutional Review Board [WIRB]). If there were unexpected adverse events, than termination of the trial would be considered in concert with the study team and WIRB.

**SPIRIT Item #23: Frequency and procedures for auditing trial conduct, if any, and whether the process will be independent from investigators and the sponsor.**

Trial conduct will be monitored by the PI in conjunction with the study team and through communication with Western Institutional Review Board (WIRB). Below, we detail how the PI and study team will monitor the trial for safety and data quality.

Monitoring of Safety: As risk for adverse events is minimal with the proposed self-report, behavioral, and MRI assessments procedures and behavioral therapy interventions, there will be no external monitoring. Data safety and monitoring will be carried out to ensure and maintain the scientific integrity of this project and to protect the safety of our participants, and will be the responsibility of the PI and Co-I team in concert with the institutional review board. Safety monitoring involves review of accumulated outcome data for groups of participants to determine if any of the procedures practiced should be altered or stopped. Dr. Aupperle (PI) will be responsible for (a) monitoring the safety of the study during weekly meetings with the study coordinator (Ms. Akeman) and clinicians (Ms. Santiago and Dr. Kirlic) and monthly meetings with Co-Investigator Dr. Paulus and (b) complying with reporting requirements. Dr. Paulus, M.D. (Co-Investigator) will be available for consultation for any medical or safety issues. Additionally, close monitoring of participant safety will include prompt reporting of adverse events to the IRB. Dr. Aupperle (PI) will provide a summary of the safe conduct of the study to NIH as part of the annual progress report, which will parallel the written report required by the IRB for annual project renewal. Review of data and procedures may result in early termination of the study, amendment to the protocol, or changes to the data collection plan. Should the protocol or data collection plans be amended as a result of data review, the IRB will be notified and the amendment approved prior to study amendment implementation. In addition, participants will be notified of any significant new risks that develop during the course of research that may affect their wish to continue study participation. Any unanticipated adverse events will be reported immediately to the Laureate Institute for Brain Research Human Protection Administrator at (918) 502-5155 or via email at hpa@laureateinstitute.organd to the Western IRB. Any adverse events will be included in the annual IRB report.

Monitoring of Data Quality: A number of procedures are in place to assure data integrity and protocol adherence. We will use Research Electronic Data Capture to support direct data entry by patients and study staff. REDCap is a free, secure, HIPAA compliant web-based application hosted locally by the Laureate Institute for Brain Research (LIBR). Web-based surveys rely on a study-specific data dictionary defined by members of the research team, with capability for systematic data querying and checking.

Monthly reports will monitor subject enrollment, completion, attrition, and individual subject progress as well as the completion of critical assessments. Additional reports will be done as needed to monitor baseline characteristics, protocol adherence, and other issues of interest. In order to ensure confidentiality, data will be identified in the database only by subject number, visit number, and date of visit. Any data that is transmitted electronically will be fully encrypted and password protected. Subjects’ names will not be entered into the database; each will be uniquely identified only by an ID number. Hardcopy data will be kept and filed in locked office cabinets.

**SPIRIT Item #26b: Additional consent provisions for collection and use of participant data and biological specimens in ancillary studies, if applicable.**

On the consent form, participants will informed that they can withdraw or take away permission to use and disclose their health information at any time. They are instructed to do this by sending written notice to the study doctor. They are informed that when they withdraw permission, no new health information identifying them will be gathered after that date. Information that has already been gathered may still be used and given to others. They are also informed that, in addition to the study investigators and study staff, individuals employed by the sponsor (Laureate Institute for Brain research) may obtain this information for use in ancillary studies, including biological specimens collected and stored. The participants are also presented with an optional consent form, providing them the opportunity to consent to use the audio and video files from therapy sessions to train clinicians external to the institute, which would involve releasing confidential data. Signing of this consent is not required for participation in the study and no audio or video files are shared outside of the sponsoring institution and study team unless all participants within a particular therapy group provide this optional consent.

Supplementary Table 3. World Health Organization Trial Registration Data Set information

| **Data category** | **Information** |
| --- | --- |
| Primary registry and trial identifying number | ClinicalTrials.gov #NCT02807480 |
|  |  |
| Date of registration in primary registry | June 21, 2016 |
| Secondary identifying numbers | N/A |
| Source(s) of monetary or material support | NIMH K23MH108707  William K. Warren Foundation |
| Primary sponsor | Laureate Institute for Brain Research |
| Secondary sponsor(s) | N/A |
| Contact for public queries | Elisabeth Akeman, eakeman@libr.net |
| Contact for scientific queries | Robin Aupperle, PhD, raupperle@libr.net |
|  |  |
| Public title | Approach-Avoidance Conflict-a Multi-level Predictor for Therapy Response |
| Scientific title | Approach-Avoidance Conflict-a Multi-level Predictor for Therapy Response |
| Countries of recruitment | United States |
| Health condition(s) or problem(s) studied | generalized anxiety disorder |
| Intervention(s) | see Intervention section of Methods |
| Key inclusion and exclusion criteria | see Participants section of Methods |
|  |  |
|  |  |
| Study type | Interventional  Allocation: randomized clinical trial, parallel assignment, evenly distributed arms.  Masking: outcomes assessor masked to intervention condition |
| Date of first enrolment | June 7, 2016 |
| Target sample size | 100 |
| Recruitment status | Recruiting |
| Primary outcome(s) | GAD-7 self-report score (time frame: post-treatment, on average at 16 weeks after baseline assessment) |
| Key secondary outcomes | Sheehan Disability Scale  NIH PROMIS Anxiety Scale  NIH PROMIS Depression Scale |

Abbreviations: NIMH: National Institute of Mental Health; NIH: National Institute of Health; GAD: generalized anxiety disorder; PROMIS: Patient-Reported Outcomes Measurement Information System.

**References**

1. Kazantzis N, Deane FP, Ronan KR. Assessing compliance with homework assignments: review and recommendations for clinical practice. J Clin Psychol. 2004;60(6):627-41. doi:10.1002/jclp.10239

2. Tarrier N, Yusupoff L, McCarthy E, Kinney C, Wittkowski A. Some reasons why patients suffering from chronic schizophrenia fail to continue in psychological treatment. Behav Cogn Psychother. 1998;26(2):177-81.

3. Ball SA, Carroll KM, Canning-Ball M, Rounsaville BJ. Reasons for dropout from drug abuse treatment: Symptoms, personality, and motivation. Addict Behav. 2006;31(2):320-30.

4. Post RM, Roy-Byrne PP, Uhde TW. Graphic representation of the life course of illness in patients with affective disorder. Am J Psychiatry. 1988;145(7):844-8. doi:10.1176/ajp.145.7.844

5. Denicoff KD, Ali SO, Sollinger AB, Smith-Jackson EE, Leverich GS, Post RM. Utility of the daily prospective National Institute of Mental Health Life-Chart Method (NIMH-LCM-p) ratings in clinical trials of bipolar disorder. Depress Anxiety. 2002;15(1):1-9.

6. MacLeod C, Mathews A. Anxiety and the allocation of attention to threat. Q J Exp Psychol A. 1988;40(4):653-70.

7. Pizzagalli DA, Jahn AL, O’Shea JP. Toward an objective characterization of an anhedonic phenotype: a signal-detection approach. Biol Psychiatry. 2005;57(4):319-27.

8. Minassian A, Henry BL, Young JW, Masten V, Geyer MA, Perry W. Repeated assessment of exploration and novelty seeking in the human behavioral pattern monitor in bipolar disorder patients and healthy individuals. PLOS one. 2011;6.8:e24185. doi:10.1371/journal.pone.0024185

9. Akintola AA, van de Pol V, Bimmel D, Maan AC, van Heemst D. Comparative Analysis of the Equivital EQ02 Lifemonitor with Holter Ambulatory ECG Device for Continuous Measurement of ECG, Heart Rate, and Heart Rate Variability: A Validation Study for Precision and Accuracy. Front Physiol. 2016;7:391. doi:10.3389/fphys.2016.00391

10. Knutson B, Westdorp A, Kaiser E, Hommer D. FMRI visualization of brain activity during a monetary incentive delay task. Neuroimage. 2000;12(1):20-7.

11. Knutson B, Adams CM, Fong GW, Hommer D. Anticipation of increasing monetary reward selectively recruits nucleus accumbens. J Neurosci. 2001;21(16):159-64.

12. Knutson B, Bhanji JP, Cooney RE, Atlas LY, Gotlib IH. Neural responses to monetary incentives in major depression. Biol Psychiatry. 2008;63(7):686-92.

13. Hariri AR, Tessitore A, Mattay VS, Fera F, Weinberger DR. The Amygdala Response to Emotional Stimuli: A Comparison of Faces and Scenes. Neuroimage. 2002;17(1):317-23.

14. Tottenham N, Tanaka JW, Leon AC, McCarry T, Nurse M, Hare TA, et al. The NimStim set of facial expressions: Judgments from untrained research participants. Psychiatric Res. 2009:1-8. doi:10.1016/j.psychres.2008.05.006

15. Cox RW. AFNI: software for analysis and visualization of functional magnetic resonance neuroimages. Comput Biomed Res. 1996;29(3):162-73. doi:10.1006/cbmr.1996.0014

16. Wong C-K, Zotev V, Misaki M, Phillips R, Luo Q, Bodurka J. Automatic EEG-assisted retrospective motion correction for fMRI (aE-REMCOR). Neuroimage. 2016;129:133.

17. Glover GH, Li TQ, Ress D. Image‐based method for retrospective correction of physiological motion effects in fMRI: RETROICOR. Magn Reson Med. 2000;44(1):162-7.

18. McLean CP, Asnaani A, Litz BT, Hofmann SG. Gender differences in anxiety disorders: prevalence, course of illness, comorbidity and burden of illness. J Psychiatr Res. 2011;45(8):1027-35. doi:10.1016/j.jpsychires.2011.03.006
