# Supplementary material for: Protocol for a randomized controlled trial examining multilevel prediction of response to behavioral activation and exposure-based therapy for generalized anxiety disorder
Source: Trials. 2020 Jan 6;21:17. doi: 10.1186/s13063-019-3802-9 (PMC6943897; doi:10.1186/s13063-019-3802-9)
Supplement: Supplementary file 4 — Additional file 4. K23 Notice of Award: Letter from the National Institute of Mental Health informing the principal investigator of the award granted for this protocol. [file 13063_2019_3802_MOESM4_ESM.pdf]

Notice of Award

MENTORED PATIENT-ORIENTED RESEARCH **Federal Award Date:** 04/12/2016

Department of Health and Human Services
National Institutes of Health

NATIONAL INSTITUTE OF MENTAL HEALTH

Grant Number: 1K23MH108707-01A1
FAIN: K23MH108707

Principal Investigator(s):
ROBIN L AUPPERLE, PHD

Project Title: Approach-Avoidance Conflict-a multi-level predictor for exposure therapy response

McCallum, Colleen
Chief Operating Officer
6655 South Yale Avenue
Tulsa, OK 741363326

Award e-mailed to: cmccallum@laureateinstitute.org

Period Of Performance:
Budget Period: 04/12/2016 – 03/31/2017
Project Period: 04/12/2016 – 03/31/2021

Dear Business Official:

The National Institutes of Health hereby awards a grant in the amount of $181,532 (see “Award Calculation” in Section I and “Terms and Conditions” in Section III) to LAUREATE INSTITUTE FOR BRAIN RESEARCH in support of the above referenced project. This award is pursuant to the authority of 42 USC 241 42 CFR 52 and is subject to the requirements of this statute and regulation and of other referenced, incorporated or attached terms and conditions.

Acceptance of this award including the “Terms and Conditions” is acknowledged by the grantee when funds are drawn down or otherwise obtained from the grant payment system.

Each publication, press release, or other document about research supported by an NIH award must include an acknowledgment of NIH award support and a disclaimer such as “Research reported in this publication was supported by the National Institute Of Mental Health of the National Institutes of Health under Award Number K23MH108707. The content is solely the responsibility of the authors and does not necessarily represent the official views of the National Institutes of Health.” Prior to issuing a press release concerning the outcome of this research, please notify the NIH awarding IC in advance to allow for coordination.

Award recipients must promote objectivity in research by establishing standards that provide a reasonable expectation that the design, conduct and reporting of research funded under NIH awards will be free from bias resulting from an Investigator’s Financial Conflict of Interest (FCOI), in accordance with the 2011 revised regulation at 42 CFR Part 50 Subpart F. The Institution shall submit all FCOI reports to the NIH through the eRA Commons FCOI Module. The regulation does not apply to Phase I Small Business Innovative Research (SBIR) and Small Business Technology Transfer (STTR) awards. Consult the NIH website http://grants.nih.gov/grants/policy/coi/ for a link to the regulation and additional important information.

If you have any questions about this award, please contact the individual(s) referenced in Section IV.

Sincerely yours,

Heather Weiss
Grants Management Officer
NATIONAL INSTITUTE OF MENTAL HEALTH

Additional information follows

SECTION I – AWARD DATA – 1K23MH108707-01A1

Award Calculation (U.S. Dollars)

**Federal Direct Costs**	$168,085
**Federal F&A Costs**	$13,447
**Approved Budget**	$181,532
**Total Amount of Federal Funds Obligated (Federal Share)**	$181,532
**TOTAL FEDERAL AWARD AMOUNT**	$181,532
**AMOUNT OF THIS ACTION (FEDERAL SHARE)**	$181,532

SUMMARY TOTALS FOR ALL YEARS		
YR	THIS AWARD	CUMULATIVE TOTALS
1	$181,532	$181,532
2	$182,428	$182,428
3	$183,811	$183,811
4	$184,221	$184,221
5	$184,761	$184,761

Recommended future year total cost support, subject to the availability of funds and satisfactory progress of the project

Fiscal Information:

CFDA Name: Mental Health Research Grants
CFDA Number: 93.242
EIN: 1731328881A1
Document Number: KMH108707A
PMS Account Type: P (Subaccount)
Fiscal Year: 2016

IC	CAN	2016	2017	2018	2019	2020
MH	8472598	$181,532	$182,428	$183,811	$184,221	$184,761

Recommended future year total cost support, subject to the availability of funds and satisfactory progress of the project

NIH Administrative Data:
PCC: AK-TN / **OC**: 415L / **Released**: WEISSHE 04/06/2016
Award Processed: 04/12/2016 12:07:06 AM

SECTION II – PAYMENT/HOTLINE INFORMATION – 1K23MH108707-01A1

For payment and HHS Office of Inspector General Hotline information, see the NIH Home Page at http://grants.nih.gov/grants/policy/awardconditions.htm

SECTION III – TERMS AND CONDITIONS – 1K23MH108707-01A1

This award is based on the application submitted to, and as approved by, NIH on the above-titled project and is subject to the terms and conditions incorporated either directly or by reference in the following:

a. The grant program legislation and program regulation cited in this Notice of Award.
b. Conditions on activities and expenditure of funds in other statutory requirements, such as those included in appropriations acts.
c. 45 CFR Part 75.
d. National Policy Requirements and all other requirements described in the NIH Grants Policy Statement, including addenda in effect as of the beginning date of the budget period.
e. Federal Award Performance Goals: As required by the periodic report in the RPPR or in the final progress report when applicable.
f. This award notice, INCLUDING THE TERMS AND CONDITIONS CITED BELOW.

(See NIH Home Page at http://grants.nih.gov/grants/policy/awardconditions.htm for certain references cited above.)

Research and Development (R&D): All awards issued by the National Institutes of Health (NIH) meet the definition of "Research and Development" at 45 CFR Part§ 75.2**.** As such, auditees should identify NIH awards as part of the R&D cluster on the Schedule of Expenditures of Federal Awards (SEFA). The auditor should test NIH awards for compliance as instructed in Part V, Clusters of Programs. NIH recognizes that some awards may have another classification for purposes of indirect costs. The auditor is not required to report the disconnect (i.e., the award is classified as R&D for Federal Audit Requirement purposes but non-research for indirect cost rate purposes), unless the auditee is charging indirect costs at a rate other than the rate(s) specified in the award document(s).

An unobligated balance may be carried over into the next budget period without Grants Management Officer prior approval.

This grant is subject to Streamlined Noncompeting Award Procedures (SNAP).

This award is subject to the requirements of 2 CFR Part 25 for institutions to receive a Dun & Bradstreet Universal Numbering System (DUNS) number and maintain an active registration in the System for Award Management (SAM). Should a consortium/subaward be issued under this award, a DUNS requirement must be included. See http://grants.nih.gov/grants/policy/awardconditions.htm for the full NIH award term implementing this requirement and other additional information.

This award has been assigned the Federal Award Identification Number (FAIN) K23MH108707. Recipients must document the assigned FAIN on each consortium/subaward issued under this award.

Based on the project period start date of this project, this award is likely subject to the Transparency Act subaward and executive compensation reporting requirement of 2 CFR Part 170. There are conditions that may exclude this award; see http://grants.nih.gov/grants/policy/awardconditions.htm for additional award applicability information.

In accordance with P.L. 110-161, compliance with the NIH Public Access Policy is now mandatory. For more information, see NOT-OD-08-033 and the Public Access website: http://publicaccess.nih.gov/.

This award provides support for one or more clinical trials. By law (Title VIII, Section 801 of Public Law 110-85), the "responsible party" must register "applicable clinical trials" on the ClinicalTrials.gov Protocol Registration System Information Website. NIH encourages registration of all trials whether required under the law or not. For more information, see http://grants.nih.gov/ClinicalTrials_fdaaa/

In accordance with the regulatory requirements provided at 45 CFR 75.113 and Appendix XII to 45 CFR Part 75, recipients that have currently active Federal grants, cooperative agreements, and procurement contracts with cumulative total value greater than $10,000,000 must report and maintain information in the System for Award Management (SAM) about civil, criminal, and administrative proceedings in connection with the award or performance of a Federal award that reached final disposition within the most recent five-year period. The recipient must also make semiannual disclosures regarding such proceedings. Proceedings information will be made publicly available in the designated integrity and performance system (currently the Federal Awardee Performance and Integrity Information System (FAPIIS)). Full reporting requirements and procedures are found in Appendix XII to 45 CFR Part 75. This term does not apply to NIH fellowships.

Treatment of Program Income:
Additional Costs

SECTION IV – MH Special Terms and Conditions – 1K23MH108707-01A1

AWARD NOTICE:
This award has been made in response to the application submitted under the Funding Opportunity Announcement PA14-049 which can be referenced at: http://grants.nih.gov/grants/guide/pa-files/PA-14-049.html.

INITIAL BUDGET PERIOD:

Although the budget period end date for this award is 03/31/2017, this award includes funds for 12-months of support. Subsequent budget periods will begin on 04/01, and will be 12-months in duration. Allowable pre-award costs may be charged to this award in accordance with the conditions outlined in the NIH Grants Policy Statement (November 2015) http://grants.nih.gov/grants/policy/nihgps/index.htm and with the recipient organization's requirements for prior approval.

DIRECT COSTS:
Please note that the direct costs recommended in all years are based on the NIMH Policy for Career (K) Awards as outlined in NOT-MH-13-001. The policy can be found at: http://www.nimh.nih.gov/funding/training/career-development-programs-k-series.shtml.

REPORTING REQUIREMENT:
Successful completion of instruction in Responsible Conduct of Research is required during Year 01 of this award (see http://www.nimh.nih.gov/funding/training/career-development-programs-k-series.shtml). Instructional details must be reported in the Year 02 Progress Report. Full details about this policy requirement can be found in the NIH Guide Notice NOT-OD-10-019 which can be accessed at: http://grants.nih.gov/grants/guide/notice-files/NOT-OD-10-019.html.

STAFF CONTACTS

The Grants Management Specialist is responsible for the negotiation, award and administration of this project and for interpretation of Grants Administration policies and provisions. The Program Official is responsible for the scientific, programmatic and technical aspects of this project. These individuals work together in overall project administration. Prior approval requests (signed by an Authorized Organizational Representative) should be submitted in writing to the Grants Management Specialist. Requests may be made via e-mail.

Grants Management Specialist: John E. Fonda
Email: john.fonda@nih.gov **Phone**: 301-402-8158 **Fax**: 301-480-1956

Program Official: Mark Chavez
Email: mchavez1@mail.nih.gov **Phone**: 301-443-8942 **Fax**: 301-480-3284

SPREADSHEET SUMMARY
GRANT NUMBER: 1K23MH108707-01A1

INSTITUTION: LAUREATE INSTITUTE FOR BRAIN RESEARCH

Facilities and Administrative Costs	Year 1	Year 2	Year 3	Year 4	Year 5
F&A Cost Rate 1	8%	8%	8%	8%	8%
F&A Cost Base 1	$168,085	$168,915	$170,195	$170,575	$171,075
F&A Costs 1	$13,447	$13,513	$13,616	$13,646	$13,686
