# Supplementary material for: Protocol for a randomized controlled trial examining multilevel prediction of response to behavioral activation and exposure-based therapy for generalized anxiety disorder
Source: Trials. 2020 Jan 6;21:17. doi: 10.1186/s13063-019-3802-9 (PMC6943897; doi:10.1186/s13063-019-3802-9)
Supplement: Supplementary file 5 — Additional file 5. Study consent form: The informed consent document approved by the Western Institutional Review Board. [file 13063_2019_3802_MOESM5_ESM.docx]

*Laureate Institute for Brain Research, Inc., Tulsa, OK, United States*

| **Research subject information and Consent form** | |
| --- | --- |
| **TITLE:** | Approach-Avoidance Conflict-a multi-level predictor for therapy response |

**This consent form contains important information to help you decide whether to participate in a research study.**

The study staff will explain this study to you. Ask questions about anything that is not clear at any time. You may take home an unsigned copy of this consent form to think about and discuss with family or friends.

- **Being in a study is voluntary – your choice.**
- **If you join this study, you can still stop at any time.**
- **No one can promise that a study will help you.**
- **Do not join this study unless all of your questions are answered.**

**After reading and discussing the information in this consent form you should know:**

- Why this research study is being done;
- What will happen during the study;
- Any possible benefits to you;
- The possible risks to you;
- Other options you could choose instead of being in this study;
- How your personal health information will be treated during the study and after the study is over;
- Whether being in this study could involve any cost to you; and
- What to do if you have problems or questions about this study.

**Please read this consent form carefully.**

**RESEARCH SUBJECT INFORMATION AND CONSENT FORM**

**Title:** Approach-Avoidance Conflict-a multi-level predictor for therapy response

**Protocol No.:** 2015-006-03

WIRB^®^ Protocol #20151232

**Sponsor:** Laureate Institute for Brain Research (LIBR)

**Investigator:** Robin Aupperle, PhD

6655 S Yale Ave

Tulsa, Oklahoma 74136

United States

**STUDY-RELATED**

**PHONE NUMBER(S):** Akalvizhy Elanko

918-344-8611

Robin Aupperle, PhD

918-502-5155

918-481-4000 (24 hours)

This consent form describes a research study. Research studies involve only individuals who choose to participate. Please take your time to make your decision about participating in the study.

This consent form may contain words that you do not understand. Please ask the Principal Investigator or the study staff to explain any words or information that you do not clearly understand. You may take home an unsigned copy of this consent form to think about or discuss with family or friends before making your decision.

A description of this clinical trial will be available on http://www.ClinicalTrials.gov, as required by U.S. Law. This Web site will not include information that can identify you. At most, the Web site will include a summary of the results. You can search this Web site at any time.

**Why Have You Been Asked To Participate In This Study?**

You are being asked to take part in this study because:

- You Have Depression

or

- You have Anxiety Disorder

**Why Is This Study Being Done?**

This project seeks to identify brain and behavioral characteristics of depressed and anxious people that will predict the effectiveness of Behavior Therapy. In addition, this project seeks to determine which of two types of behavior therapy may be most useful for individuals diagnosed with generalized anxiety disorder. Brain imaging experiments will use functional magnetic resonance imaging (fMRI) brain scanning. The MRI scanner measures changes in blood oxygen levels while you perform mental tasks. This procedure involves no injections or radiation and will provide information about which brain areas become more active (that is, receive more blood oxygen) while you are performing the tasks. Behavior Therapy will consist of weekly group therapy sessions targeting either depression or anxiety symptoms.

**What Devices Are Involved In This Study?**

The Magnetic Resonance Imaging (MRI - use of a magnetic field to produce an image) scanner is used to look at brain function and anatomy. The LIBR is using the MRI scanner in a research (experimental) mode. U.S. Food and Drug Administration (FDA) approval has not been obtained for the particular ways that information about inter-personal interaction from the MRI may be used in this research; however, the MRI scanner will not be used for purposes outside its intended application. It is considered to be a non-significant risk investigational device.

A physiological recording system will also be used to monitor bodily responses such as heart rate and breathing.

**How Many Subjects Will Take Part In The Study?**

About 150 subjects will take part in this study at the Laureate Institute for Brain Research

**What Is Involved In The Study?**

If you take part in this study, you may have the following tests and procedures at the Laureate Institute for Brain Research:

*Interview, Questionnaires & Behavioral Tests*

You will be asked to complete written tests. You will also sit in front of a computer and make decisions about what you are shown. These tests and questionnaires about your health will measure your mental and physical states. You will have an interview with a member of the research staff, where you will be asked a series of questions about your mental and physical health, sexual practices and function, substance abuse and symptoms you may have experienced during your lifetime. See the Visit Schedule below for the specific times for each visit.

For the behavioral testing, you will be fitted with a vest with motion sensors and you will be asked to perform certain tasks. We may video and audio record you while you complete some of the behavioral tests, which will allow us to measure emotional expressions and behaviors. It will only be viewed by the research team and will be destroyed when the study is closed. Computer behavioral tasks will involve making decisions in response to various potential rewards (for example, points) or risks (for example, losing points or viewing negative images and sounds). The images and sounds used will include emotional faces or emotional images. The negative images and sounds may include such things as dead bodies, injury, or blood. The positive images and sounds may include such things as families laughing or nature scenes. You will be responding to these various tasks using button presses or joystick movements.

*Electroencephalography (EEG)*

You may also be asked to have an EEG at the same time as the MRI scan. After you are familiarized with the equipment, a cap consisting of multiple MRI-compatible EEG electrodes with gel in them will be placed on your scalp and a few electrodes with gel in them will be placed on your back. While you lie in the MRI scanner, the electrodes will be connected to a data collection system and EEG signals will be collected and stored on the computer of the recording system. The prep time for an EEG takes approximately an hour.

*Magnetic Resonance Imaging (MRI)*

For MRI studies, you will be given a brief medical history questionnaire and screening form to complete. Undesirable medical findings may arise during the interview, screening, or MRI scanning. If so, these findings will remain confidential and will be discussed with you by a researcher of this study.

Before MRI scanning, you will learn tasks that will involve (a) responding to positive and negative emotional images and sounds, (b) making decisions when faced with potential emotional or rewarding outcomes, (c) responding or not responding to images, or (d) pay attention to sensations in your body (i.e., heart or stomach). Training for the MRI tasks will last approximately 15 minutes. You will then be placed in the MRI scanner to perform the tasks. The MRI scanner rapidly takes pictures of your brain. The MRI scanner is a metal tube surrounded by a strong magnetic field. During the MRI, you will lie on a table that can slide in and out of the tube. You will be asked to lie still during scanning. Earplugs will be provided to lessen the loud “knocking” sounds that the scanner makes while it is imaging your brain.

First, you will receive a series of short scans that allow us to know where your head is inside the tube. You will then receive a scan lasting 5 to 15 minutes that gives us more detailed pictures of your brain. Finally, you will undergo a series of scans lasting about 60 minutes during which you will perform the tasks. During all of these scans, it will be very important to remain still. Your time in the scanner will be about 1.5 hours.

If you are a female, a urine pregnancy test will be obtained. An over-the-counter urine pregnancy test will be completed just prior to any MRI scanning. You will not be allowed to participate in the study if the pregnancy test reads positive.

*Blood Sampling*

A needle will be used to draw blood from a vein in your arm by a registered nurse or trained phlebotomist. A total of about 200 mL (less than 14 tablespoons) of blood is drawn during the entire study. Sometimes a blood test may need to be repeated. If this happens the total amount of blood drawn will be more than this. The blood is being drawn to investigate potential metabolic and inflammatory biomarkers, or genetic information, that may predict response to Behavior Therapy.

*Physical Measurements*

We will measure your vital signs (blood pressure and pulse), your height, weight, body fat, and measurement of your hip and waist. You may be asked to provide a breath sample to test for recent alcohol use and a urine drug test.

*Treatment Visits*

You will be asked to participate in 10 group sessions that will last about 1 ½ hours each. For most participants, your treatment will be specific for your diagnosis. However, if you meet criteria for generalized anxiety disorder (GAD), you will be randomized to receive Behavioral Activation or Exposure Therapy. Randomization means that you are put into a group by chance. A computer program will place you in one of the study groups. Neither you nor your study doctor can choose the group you will be in. You will have an equal chance of being placed in either group.

All therapy sessions will be led by a licensed Clinical Social Worker, licensed Clinical Psychologist, and/or a clinical psychology doctoral student. Each therapy session will be video and audio recorded and may be reviewed by the research team. The recordings will be destroyed by the end of the study, unless consent is given to use them for educational and training purposes (see consent form addendum).

The table below is an estimate of the amount of time it will take to complete all visits and the payment per visit. If you withdraw from the study prior to finishing treatment, you will also be asked to complete a set of assessments via phone or online, estimated to take approximately 10-15 minutes.

**Visit Schedule**

|  | Pre- Treatment Visit | Weekly Treatment Visits 1-10 | Post- Treatment Visit | 3-month Follow Up | 6-month Follow Up |
| --- | --- | --- | --- | --- | --- |
| Clinical & Self-Report Questionnaires and Behavioral tasks | 7 hours | 15 minutes | 7 hours | .5 hours | .5 hours |
| MRI Scan | 2.5 hours | -------- | 2.5 hours | ---------- | ---------- |
| Treatment Session | -------- | 1 ½ hours | --------- | ---------- | ---------- |

In addition to the in-person therapy sessions, you will be provided between-session assignments to complete as part of that therapy. You may also be contacted by clinicians or research staff in between sessions for scheduling purposes or to check in regarding therapy assignments.

**How Long Will You Be In The Study?**

You will receive treatment therapy once a week for 10 weeks. You will also have a pre and post treatment study visit. Your time in this study will be about 12-20 weeks for the main part of the study or approximately 40-45 weeks for completion of both 3- and 6- month follow-up surveys.

There may be circumstances under which your participation in the study may be stopped by the investigator without your consent. Functional MRI is dependent upon measuring very small changes in blood flow in the brain. Therefore, there may be times in which the information collected will be unusable due either to a scanner malfunction or from you moving your head too much. Under these circumstances, your participation may be stopped without your consent.

***You may stop participating in this study at any time.***

**What Are The Risks Of The Study?**

The study may involve the risks described in this section.

It is possible that your participation in this study might reveal unanticipated medical or psychiatric findings. You have the right to refuse answering any question that you do not wish to answer and to withdraw from participating in the study at any time.

This study is being done identify brain and behavioral characteristics of depressed and anxious people that will predict the effectiveness of Behavior Therapy. The types of Behavior Therapy being utilized for depression (Behavioral Activation) and anxiety (Exposure Therapy) have been shown by previous research to reduce mood and anxiety symptoms for some people. However, it is possible that medical or psychiatric problems may arise or be identified during the screening process, for which we may not be able to provide treatment. It is important that you understand that treatment for medical or psychiatric conditions other than mild to moderate depression or anxiety will not be offered in this study.

Individuals who meet criteria for generalized anxiety disorder (GAD) will be randomized to receive Behavioral Activation or Exposure Therapy. There is reason to believe that either of these therapies may be effective in improving symptoms associated with generalized anxiety disorder and/or associated mood symptoms for some people. However, it is possible that the treatment you are randomized to receive may not be useful for your symptoms.

If unanticipated findings occur, we will provide you with all results of screening tests and will assist you in communicating these results to your primary physician or other health care provider, so long as you provide written consent for us to do so.

The risks of the study procedures are listed and discussed below.

**Risks Associated with Interview, Questionnaires, & Behavioral Testing**

The interview and questionnaires about your health, mood, and behavior and the behavioral tests are not physically harmful but may be stressful to complete and may be sensitive and emotionally distressing. In particular, the behavioral tests may include distressing images that include such things as dead bodies, blood, and war. We ask only that you try your best. You may stop any test at any time.

**Risks Associated with Motion Sensor Vest**

There are no significant risks expected from the vest. However, there may be minor discomfort from wearing the vest.

**Risks Associated with EEG**

There are no significant risks expected from the MRI-compatible EEG. However, there may be minor discomfort from wearing an EEG cap, which is similar to wearing a tight hat. You may feel local pressure points and minor skin irritation from the electrode patches. This will be minimized by the use of gel in each electrode, and foam pads placed under and surrounding your head.

**Risks of MRI scanning**

People are at risk for injury from the MRI magnet if they have any of the following metal implants or fragments:

- pacemakers or other implanted electronic devices
- brain stimulators
- dental implants
- aneurysm clips (metal clips on the wall of a large artery)
- metallic prostheses (including metal pins and rods, heart valves, and cochlear implants)
- permanent eyeliner
- implanted delivery pump
- shrapnel fragments

You will be asked to complete an MRI screening form for the MRI scan. You will be screened for these implants or metal fragments before the study, and if you have any of them, you will not receive an MRI scan and cannot be in the study. Tell the study doctor if you are uncertain whether you have any metal objects in your body. In addition, all magnetic objects (for example, watches, coins, jewelry, and credit cards) must be removed before entering the MRI scanner room.

Welders and metal workers are also at risk for injury because they may be unaware of small metal fragments in the eye.

There are no known long-term risks or consequences of MRI scans. However, you may become uncomfortable because you will be lying in a small space. Some people are bothered by the loud thumping noises made by the scanner. You will wear earplugs to reduce the noise and increase your comfort during scanning. LIBR study staff will closely and continuously monitor you throughout the scanning procedure. You will be able to use an emergency call button at all times during the scan. You will be removed from the scanner immediately if you request to be removed.

**Risks associated with Blood Draws**

Taking blood may cause some pain, discomfort, bleeding, or bruising/swelling where the needle enters the body, and, in rare cases, dizziness, lightheadedness, fainting, or infection.

**Genetic Testing Risk**

A Federal law, called the Genetic Information Nondiscrimination Act (GINA), provides some protection for your genetic information. However, this Federal law does not protect you against genetic discrimination by companies that sell life insurance, disability insurance, or long-term care insurance. This law generally will protect you in the following ways:

- Health insurance companies and group health plans may not request your genetic information collected in this research.
- Health insurance companies and group health plans may not use your genetic information when making decisions regarding your eligibility or premiums.
- Employers with 15 or more employees may not use your genetic information collected in this research when making a decisions about your employment.

**Risks for Females**

If you are a female, you must not be pregnant while participating in this study. A urine-based pregnancy test will be obtained prior to any MRI scanning.

**Risks for Group Therapy Sessions**

There is minimal risk in participating in the group therapy sessions. However, your depression or anxiety could increase over time in the study. You will be monitored weekly for a change in your symptoms by researchers. A clinical psychologist and psychiatrist are part of the research team.

**Are There Benefits To Taking Part In The Study?**

The types of Behavior Therapy being utilized for depression (Behavioral Activation) and anxiety (Exposure Therapy) in this study have been shown by previous research to reduce mood and anxiety symptoms for some people. Therefore, there is a possibility that you will benefit from this treatment. In addition, your participation should help us understand what behaviors and brain responses may predict how individuals respond to these treatments. Understanding what predicts therapy response could help us develop additional treatments for mood and anxiety disorders.

**What Are The Costs Of Participating In The Study?**

Neither you nor your health insurance will be charged for any of the study tests, procedures, or activities. If you need to be hospitalized, voluntarily or involuntarily, Laureate Institute for Brain Research does not intend to provide payment for this, and you or your insurance provider will be billed for these costs.

**Will You Be Paid For Participating In This Study?**

For the interview, questionnaires, and the behavioral tests, you will be paid $10 per half hour. While you are prepped for the EEG and when you are in the MRI scanner, you will be paid $25 per half hour. Your time in the MRI scanner will be approximately 1 ½ hours. For blood draws, you will be paid $50 per half hour. You may earn $0-55 more based on your participation in the decision-making task. If you withdraw from the study prior to completing treatment, you will be asked to complete a set of questionnaires via phone or online, taking approximately 10-15 minutes to complete, for which you will be compensated $10.

|  | Pre-Treatment Visit | Weekly Treatment Visits 1-10 | Post-Treatment Visit | 3-month Follow Up | 6-month Follow Up |
| --- | --- | --- | --- | --- | --- |
| Clinical & Self-Report Questionnaires and Behavioral tasks | 7 hours: $140 | 15 minutes: ($5 each for 10 visits) | 7 hours: $140 | .5 hours: $20 | .5 hours:  $20 |
| EEG preparation and MRI Scan | 2.5 hours: $125 | -------- | 2.5 hours: $125 |  |  |
| Blood draw | ½ hour: $50 |  | ½ hour: $50 | -------- | -------- |
| Treatment Session | -------- | 1 ½ hours | --------- | -------- | -------- |
| **Per visit payment** | $315 | $50 | $315 | $20 | $20 |
| Possible Extra Earnings | $0-55 | -------- | $0-55 | -------- | -------- |

You will be paid through a ClinCard (similar to a debit card) that may be used 48 hours after the visit. The compensation for the pre-treatment, post-treatment, withdrawn, and 3-month and 6-month follow-up visits will be provided within 48 hours after completion of those visits. Compensation for the weekly treatment visits will be provided after completion of your last treatment session. However, compensation for the weekly treatment visits can be provided within 48 hours after each individual session if requested.

**What Other Options Are There?**

This study is for research purposes only, so your other option is not to participate in the study.

While the treatments being provided as part of this study have been found to reduce mood and/or anxiety symptoms, there are other options to obtain treatment. Your options may include approved medications, as well as the same or different behavioral therapies than is used in the current study. Information concerning community clinics and treatment resources will be provided to you as requested.

**Confidentiality**

**What information may be used and given to others?**

The study doctor will get your personal and medical information. For example:

1. Research records
2. Records about phone calls made as part of this research
3. Records about your study visits.

**Who may use and give out information about you?**

The study investigator and the study staff.

**Who might get this information?**

The sponsor of this research. “Sponsor” means any persons or companies that are:

1. working for or with the sponsor, or
2. owned by the sponsor.

**Your information may be given to:**

1. The U.S. Food and Drug Administration (FDA)
2. Department of Health and Human Services (DHHS) agencies
3. Western Institutional Review Board^®^ (WIRB^®^)

**Why will this information be used and/or given to others?**

- to do the research,
- to study the results, and
- to make sure that the research was done right.

If the results of this study are made public, information that identifies you will not be used.

**What if I decide not to give permission to use and give out my health information?**

Then you will not be able to be in this research study.

**May I review or copy my information?**

Yes, but only after the research is over.

**May I withdraw or revoke (cancel) my permission?**

Yes, but this permission will not stop automatically.

You may withdraw or take away your permission to use and disclose your health information at any time. You do this by sending written notice to the study doctor. If you withdraw your permission, you will not be able to stay in this study.

When you withdraw your permission, no new health information identifying you will be gathered after that date. Information that has already been gathered may still be used and given to others.

**Is my health information protected after it has been given to others?**

The information you provide as part of this study will be kept confidential within Laureate Institute for Brain Research, within the limits of the law. However, there is always a risk that your information will be given to others without your permission and may no longer be protected.

If you have any questions or concerns about your privacy rights, you should contact the LIBR Privacy Officer at 918-502-5155 or via email at privacy@laureateinstitute.org.

You have the right to access the medical information that has been collected about you as a part of this research study. However, you may not have access to this medical information until the entire research study is finished.

**What If You Are Injured While Participating In This Study?**

If you get hurt or sick from participating in this study, emergency medical treatment is available. In an emergency, call 911. Be sure to tell the emergency staff and other healthcare providers of your participation in this study. Contact Robin Aupperle, PhD, as soon as possible at 918-502-5155 or 918-481-4000 (24 hours) if you think you have a research-related injury or illness. You or your health insurance provider will be billed to cover the cost of the medical or emergency services provided. No funds have been set aside by the Laureate Institute for Brain Research to compensate you if you are hurt or get sick. However, you still have the right to bring a law suit if you think you were harmed and deserve compensation.

You do not give up any of your legal rights by signing this consent form.

**Who Will Provide Funding For The Study?**

Funding for this research study will be provided by the National Institute of Mental Health and by the Laureate Institute for Brain Research.

**What Are Your Rights As A Participant?**

Taking part in this study is voluntary. You may choose not to participate. Refusal to participate will involve no penalty or loss of benefits to which you are otherwise entitled. If you agree to participate and then decide against it, you can withdraw for any reason and leave the study at any time without penalty or loss of benefits to which you are otherwise entitled.

Your participation in this study may be stopped at any time by the study doctor or the sponsor without your consent for any reason, including the following:

- - if it is in your best interest
  - if you do not consent to continue in the study after being told of changes in the research that may affect you

**What If There Are New Findings?**

We will provide you with any significant new findings developed during the research study that may affect your health, welfare, or willingness to continue your participation in this study.

**Who Should You Call If You Have Questions Or Problems?**

Your contact person for this study is Akalvizhy Elanko. She can be reached during business hours at 918-502-5155 or 918-344-8611.

If you have questions, about your participation in this study, concerns, or complaints about the study, or have a research-related injury, contact the study investigator Robin Aupperle, PhD, at 918-502-5155 or 918-481-4000 (24 hours). For emergencies, call 911.

If you have questions about your rights as a research subject or if you have questions, concerns, input, or complaints about the research, you may contact:

Western Institutional Review Board^®^ (WIRB^®^)

1019 39th Avenue SE Suite 120

Puyallup, Washington 98374-2115

Telephone: 1-800-562-4789 or 360-252-2500

E-mail: Help@wirb.com

WIRB is a group of people who perform independent review of research.

WIRB will not be able to answer some study-specific questions, such as questions about appointment times. However, you may contact WIRB if the research staff cannot be reached or if you wish to talk to someone other than the research staff.

Do not sign this consent form unless you have had a chance to ask questions and have received satisfactory answers to all of your questions.

If you agree to be in this study, you will be given a signed and dated copy of this consent form.

**Signature**

I have read the information in this consent form. I have been given an opportunity to ask questions. All my questions about the study and my participation in it have been answered.

**I Agree To Participate In This Study**

I authorize the use and disclosure of my health information to the parties listed in the authorization section of this consent for the purposes described above.

Printed Name of Participant

**Consent Signature**

PARTICIPANT SIGNATURE (18 years and older) Date

SIGNATURE OF PERSON CONDUCTING INFORMED CONSENT Date

DISCUSSION
